# Adaptive user modelling in car racing games using behavioural and physiological data

**DOI:** 10.1007/s11257-017-9192-3

**Published:** 2017-05-02

**Authors:** Theodosis Georgiou, Yiannis Demiris

**Affiliations:** Personal Robotics Laboratory, Department of Electrical and Electronic Engineering, Imperial College London, Exhibition Road, South Kensington, London, SW7 2BT UK

**Keywords:** Personalised gaming, User modelling, Physiological models, Machine learning, User study, Theory of flow, Car racing game, Adaptive modelling

## Abstract

Personalised content adaptation has great potential to increase user engagement in video games. Procedural generation of user-tailored content increases the self-motivation of players as they immerse themselves in the virtual world. An adaptive user model is needed to capture the skills of the player and enable automatic game content altering algorithms to fit the individual user. We propose an adaptive user modelling approach using a combination of unobtrusive physiological data to identify strengths and weaknesses in user performance in car racing games. Our system creates user-tailored tracks to improve driving habits and user experience, and to keep engagement at high levels. The user modelling approach adopts concepts from the Trace Theory framework; it uses machine learning to extract features from the user’s physiological data and game-related actions, and cluster them into low level primitives. These primitives are transformed and evaluated into higher level abstractions such as *experience*, *exploration* and *attention*. These abstractions are subsequently used to provide track alteration decisions for the player. Collection of data and feedback from 52 users allowed us to associate key model variables and outcomes to user responses, and to verify that the model provides statistically significant decisions personalised to the individual player. Tailored game content variations between users in our experiments, as well as the correlations with user satisfaction demonstrate that our algorithm is able to automatically incorporate user feedback in subsequent procedural content generation.

## Introduction

Computer games have become an integral part of modern leisure-time. There is intense competition among game companies as they are being faced with challenges to retain user engagement in a saturated market. Steels (2004), based on the work done by Csikszentmihalyi (2000), suggests that for an activity to be self-motivating or “autotelic”, there must be a balance between task challenge and the person’s skill. Estimating the skills of the player and adapting the game challenge accordingly can lead to more engaging and immersing games.

Serious games, and in particular simulators, offer a medium for training and evaluating individuals in high risk scenarios, including for example flying, driving or performing surgery. Since individuals differ in terms of skills and preferences, a variety of training methods should be adapted to maximise training outcomes. In tasks where the end goal is similar between individuals ( for example, successfully tackling a sharp turn with high speed in a car racing game), people with less experience will need more assistance to reach the desired level. As we will clarify later, we relate this amount of assistance to the user’s Zone of Proximal Development (ZPD) (Vygotsky 1978), and we use it to estimate the challenge that a game will pose to a user. If the challenge level is higher than the user skill, this might result into increased user anxiety. On the other hand, if the user skill is higher than the game challenge this might result in increased user boredom.

In this paper, we focus on learning a user (or player) model using a combination of behavioural and physiological data. The model infers the current user’s experience, attention and performance from combinations of extracted features while playing a car racing game. We monitor these abstractions, update the user model and provide decision adjustments for the alteration of the racing track according to the user. We propose an algorithm that monitors the user performance and modifies the game experience in real-time, with the purpose of maintaining high player satisfaction and enhancing the learning process.

**Fig. 1:**
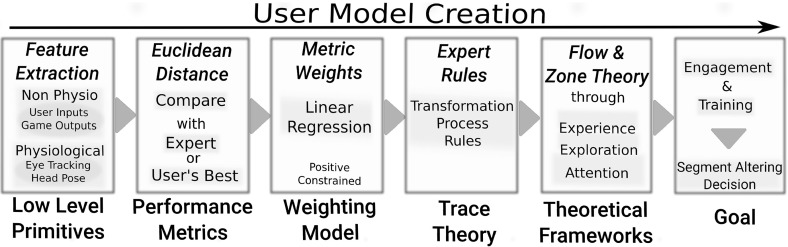
An overview of our personalised user modelling approach for evaluating a driver in car racing. Low level inputs are being converted to performance metrics using either the expert or the user’s “best” data. They are then transformed using Trace Theory through game related rules and significance weights into values representing variables from the concept of *flow*. Finally, the performance and state of the user, that is represented by these variables, are exploited to instruct how each segment path is going to be altered

As shown in Fig. 1, the proposed adaptive user model is constructed in sequential abstract layers following the Trace Theory framework (Settouti et al. 2009). Several machine learning techniques, such as Affinity Propagation (Frey and Dueck 2007) and Random Forests (Breiman 2001) are utilised to process the incoming data and transform them into available metrics, coupled with a weighting model (such as linear regression) that specifies their significance to the particular user. Finally, the higher layers are built up using ideas from educational theoretical frameworks such as the concept of *flow* (Csikszentmihalyi 1990), Zone Theory (Valsiner 1997) and the Zone of Proximal development (Vygotsky 1978); these layers provide path altering decisions for the particular user.

Our experiments are focused on the engineering and user modelling challenges underlying the detection of the optimal level of adaptation for each individual. We validate the model’s outputs against the performance and feedback data from 52 users. We conducted a user profiling analysis in order to verify and find the patterns emerging from our user responses and determine our user types.

The purpose of this article is to build a user modelling framework that triggers the alteration of the path of the track in a way that fits the skills and weaknesses of the driver. However, the algorithm of changing the track and the real human evaluation of the new segments is outside the scope of this article. In this article we studying the feasibility of the proposed approach by correlating features of the framework to the user responses.

### Background and motivation

A well-designed computer game can promote engagement and provide an effective learning environment (Whitton 2011). The perception of being good at an activity and the perception of rapid improvement both contribute positively to user engagement (Whitton 2011).  Coyne (2003) analysed the design and characteristics of various existing games and found repetition as one of the main factors of engaging games, which is usually concealed through variation either in the form of difficulty levels (new opponents, track, etc.) and/or through a narrative. Such games are based on “variation across repetitive operations” where repetition lulls the user into expectations which are subsequently challenged to enhance the user’s engagement. In our car racing game, the driving task itself is repetitive with variation introduced in the form of new tracks. The challenge that arises is customising the progression of the chosen tracks to suit the abilities of each user. Several authors have called for a balance between task difficulty and skill (Steels 2004; Demiris 2009), so that the user remains in a cognitive optimal (*flow*) state, avoid sensory overload, and remain highly engaged (Whitton 2011; Koster 2013).

Our user model aims to adapt the game challenge—path of the track—according to three concepts adopted from the concept of *flow* (Csikszentmihalyi 1990): Experience, Exploration and Attention.

- *1. Experience* estimates the skill level of the user through the user’s performance in the game. The value is determined from the average proximity of the user’s model characteristics to those of the *expert*.
- *2. Exploration* quantifies the level of consistency that the user is displaying in his/her game actions, i.e. how varied, or probing, are his current set of actions. Actions can include taking different racing paths, eye fixations, operating the interface in a different manner, among others. High values for this concept indicate that the user is finding the current task challenging, and is exploring suitable game options. Low values indicate that the user does not vary his/her actions, and has settled to a low level of variation. The reasoning behind this is that the user tries to overcome the presented challenge by attempting a new action and therefore improving their skills either through positive or negative result.
- *3. Attention* quantifies the continuous engagement of the user. This notion is based on the assumption that the *expert*’s model, from which the user features are compared, represents a fully engaged user according to *physiological* and *non-physiological* features. The attention of the user is based on the game metrics. It assumes that the user is engaged if s(he) is doing well in the game or tries to see if the physiological data are coherent to the user’s game model. To determine the attention metric, we first evaluate the average proximity (*experience*) of the user to the *expert* model using only *non-physiological* data. Getting high values from user input and game output metrics shows that user is performing well with respect to segment times and racing lines; therefore, *attention* should be high. This is based on the assumption that in order for the user to accomplish high *non-physiological* (game related) values, the user should be highly engaged in the game. Otherwise *attention* is evaluated from the current variations (*exploration*) of the user’s *physiological* data. Since the data are relative to those of the expert’s, positive *physiological exploration* means that the current features of the user are closer to the *expert*’s.

The main assumption underlying the implementation of these three concepts is that we are considering the *expert* model, which the user is compared with, as optimal in respect to engagement and performance levels. It is what the user should imitate and achieve, whereas any deviations from that are conveyed as lack of skill, challenge or attention. It is also important to mention that the model uses both physiological features from external sensors and behavioural data from the game and calculates the significance of each feature obtained according to the time performance of the user in a path. Merging the data from both domains gives the model more potential to explain the events that are unfolding in the game. Multimodal player models have been reported to be more accurate and match the user’s responses better than corresponding models built on unimodal data (Yannakakis 2009; Yannakakis et al. 2013). For example, an incorrect sequence of input actions can explain why the user crashed over a sharp turn and as a result segment’s time was poor. However, this might also have been a consequence of the user’s lack of experience in identifying the correct speed and position to brake and steer or lack of attention. The latter can only be interpreted through the concurrent monitoring of head pose and eye gaze. Doshi and Trivedi (2012) demonstrated the evoking of different pattern dynamics in eye gaze and head pose between sudden visual cues and task-oriented attention shifts.

These three high level concepts monitor the user experience while playing the game and can describe the state and engagement of the user with the game according to the combination of their values. Based on the theory of *flow* (Csikszentmihalyi 1990) there has to be balanced between challenge level and user skills, whereas attention determines how much these values are sensitive to each other. As a result, there are four main hypotheses that are possible for the particular task:

1. Both *Experience* and *Exploration* are High: This is the optimal state. Since *experience* is high, the user is engaged with the task and begins to learn the particular path. However, *exploration* is also high, therefore the user has space for more self-improvement. This means that the user’s skills are above a threshold value but not as close to the expert’s; high *exploration* indicates that the user hasn’t reached the optimal steady values of the expert’s yet. According to the interpretation of skill development by Valsiner (1997), the user is discovering a better way to tackle a path, however, this is not yet embraced as part of his/her *experience*.
2. High *Experience*, Low *Exploration*: The user is performing well; the low value of *exploration* is indicating that the user found an optimal way to handle a path and this has been adopted into the user’s skills. In order to keep the user engaged, the level of difficulty should be raised so as to challenge the user.
3. Both *Experience* and *Exploration* are Low: The user is lacking the skills for the challenge faced. Therefore, the *attention* value will be consulted to determine if the user is getting bored and giving up (low value) or if the user is engaged with the task through self-motivation to succeed (high value).
4. Low *Experience*, High *Exploration*: Challenges are much greater than the skills of the user. The user is performing poorly even when different techniques are being tried. If this state continues to persist then user’s anxiety level will increase; therefore, in order to push the user back in the engagement-training region we should drop the difficulty level of the game.

Based on the calculated values of the notions and the hypotheses, the model outputs decisions on whether a path should become easier, challenging or kept the same.

## Related research

Player modelling in games has received a lot of interest in recent years. The primary goal of player modelling is to understand the interaction of the player experience at an individual level. This can be either cognitive, affective or/and behavioural. There have been many different approaches for the understanding of a player in games (Yannakakis et al. 2013). Research is split between two areas: player modelling and player profiling. The former tries to model complex phenomena during gameplay whereas the latter tries to categorise the players based on static information, like personality or cultural background, that does not change during gameplay. Profiling is usually performed through the use of questionnaires (e.g. Five Factor Model of personality (Costa Jr and McCrae 1995), demographics) and information collected through that method can lead to a construction of better user models.

Player modelling is further split into three approaches:

1. The model-based approach: involves the mapping of user responses to game stimuli through a theory-driven model.
2. The model-free approach: assumes that there is a relation between the user input and the game states, but the underlining structure function is unknown.
3. The hybrid approach: which is the one we embraced, contains methods from both model approaches mentioned.

Game metrics, defined as statistical spatiotemporal features, are a significant component of player modelling. When these metrics are the only data available, they don’t provide sufficient information about individual users and can infer erroneous states when coming only from the game context. This can be avoided by getting feedback from users, either directly using user annotations or indirectly through sensors (e.g. cameras, eye trackers, etc.).

User annotations can be done through questionnaires or third-person reports. These are mainly of three types:

1. Rating-based format using scalar/vector values [e.g. The Game Experience Questionnaire (IJsselsteijn et al. 2008)].
2. Class-based format using Yes/No questions (Boolean).
3. Preference-based format by contrasting the user experience between consecutive gaming sessions.

In addition to the user annotation methods reviewed by Yannakakis et al. (2013) there are other methods such as think-aloud protocols for continuous annotation (Wolfe 2008) that have been used in other studies. However, those might interfere with the user engagement and can potentially become obtrusive to the game experience, so we do not use them in this paper.

### Physiological user modelling

Analysis of physiological patterns during game play has been a vital technique to assess the engagement and enjoyment of the player. Kivikangas et al. (2011) reviews a comprehensive list of references in the area of psychophysiological methods for game research. They emphasise that a commonly accepted theory for game experience does not currently exist with game researchers frequently using theoretical frameworks from other fields of study.

Similar to our concept, Tognetti et al. (2010a) presented a methodology for estimating the user preference of the opponent skill from physiological states of the user while playing a car racing game. They recorded different physiological data [e.g. heart rate (HR), galvanic skin response (GSR), respiration (RESP), temperature (T), blood volume pulse (BVP)] during each game scenario and then classified them, using Linear Discriminant Analysis (LDA), according to the user’s “Boolean” responses; their questionnaire consisted of a pairwise preference scheme (2-alternative forced choice answers) (Yannakakis and Hallam 2011). They concluded that HR and T gave poor performance on classifying the users’ emotional state where the rest (mostly GSR) achieved high correlations against user feedback. An interesting side result was that most of the users preferred an opponent of similar skills, whereas the rest were not consistent with their responses. This shows that levels of difficulty in the game are hard to pre-set for each user, and a more adaptive approach should be explored.

Similarly, Yannakakis and Hallam (2008) investigated the relationship of physiological signals (e.g. HR, BVP, GSR) with children’s entertainment preferences in various physical activity games, by utilising artificial neural networks (ANN). Through the accuracy of their ANNs on specific features (e.g. maximum, minimum, average) of the recorded signals, they demonstrated that there was a correlation between the children’s responses and the signals. They concluded that when children were having “fun”, they were more engaged displaying increased physical activity and mental effort. In addition, Yannakakis et al. (2010) investigated the effect of camera viewpoints (distance, height, frame coherence) in a PacMan-like game using physiological signals (e.g. HR, BVP, GSR) and questionnaires about the affective states of the user (fun, challenge, boredom, frustration, excitement, anxiety, relaxation). Statistical analysis of the data obtained showed that camera viewpoint parameters directly affected player performance. Emotions were correlated with HR activity (e.g high significant effects between average and minimum HR versus fun; time of minimum HR versus frustration).

Defining the level of immersion and affective state of the user in a game has been approached through different techniques. Brown and Cairns (2004) carried out a qualitative research for understanding the concept of immersion in games, by interviewing their subjects. Respectively, Jennett et al. (2008) performed three experiments on quantifying the immersion of the users in games subjectively and objectively. In the first experiment, the subject switched from a “real-world” physical task to an immersive computer game or simple mouse click activity (control group) and then back to the task. They concluded that the group playing the immersive game improved less on the time taken on carrying out the “real-world” task when compared with the control group. The explanation given by the authors was this was due to the fact that the game decreases one’s ability to re-engage in reality. The second experiment involved the investigation of eye fixation with immersion from users completing either the non-immersive task and the immersive one from the previous experiment. Self-reports revealed that there was a significant difference between the immersion level results of the two tasks, therefore, the questionnaires were a good indicator of immersion. In addition, eye gaze fixations per second increased over time for the non-immersive task group where it decreased over time on the immersive one. This shows that the control (non-immersive task) group was getting distracted more easily in irrelevant areas whereas the experiment group increased their attention in areas more relevant to the game. As we will show later in Sect. 3.4.2, the eye gaze fixations, blinking rate as well as main eye gaze positions are being utilised as features by the user model. The third experiment explored the user’s interaction and emotional state through different modes of the simple mouse clicking task (non-immersive). Through these modes the pace of the appearing box to be clicked was changing. The results showed that emotional involvement is correlated to immersion where sometimes emotion can be negative as well (e.g. anxiety in the increasing pace mode).

### User generated adaptive content and training

According to Yannakakis et al. (2013) “future games are expected to have less manual and more user-generated or procedurally-generated content”. This allows the potential for novel and enjoyable game content that is personalised to the player. Charles et al. (2005) reviews the current approaches towards player-oriented game design, examines the research conducted for better understanding and effective modelling of players and proposes a general framework that can adaptively and continuously alter the game to fit the model of the player. In order to personalise you must first understand the player through a particular game. This involves knowing the game *Mechanics* (the components and rules of the game), that give rise to game *Dynamics* (how mechanics behave on user inputs) and fuses to game *Aesthetics* (user experiences invoked by the game). Hunicke et al. (2004) refer to this as the MDA framework and show how the seamless attributes of each property relate to each other in a variety of games.

According to Malone (1980), the characteristics of an enjoyable game are: challenge, fantasy and curiosity. In an extension of the work, there was the addition of the *control* factor, empowered either by the actions available to the user, actions already taken or the potential of a great effect of the user’s decisions (Malone and Lepper 1987). In racing games the generation of content mainly targets the track’s path. Pushing the car to the limits and handling tight turns at high speeds is what engages the users in that category of games. Loiacono et al. (2011) derived an algorithm for generating new tracks in a car simulator using single and multi-objective genetic algorithms. Through certain constraints (e.g. path curvature radius, closed tracks, etc.) and the use of polar coordinates, their algorithm fills the path through particular “control” points that the road needs to cross. Cardamone et al. (2011) proposed a framework for advancing the Loiacono et al. (2011) algorithm through a human-assisted generation of tracks. Subjects voted for each generated track using scoring interfaces (5 Likert scale or Boolean type) that were influencing the algorithm over the next generations of tracks. They showed that there was an improvement of user satisfaction in younger generations.

A similar approach for user-oriented track generation was proposed by Togelius et al. (2006, 2007). They used a neural-network based controller (Togelius and Lucas 2006), that was trained on human driver behaviour, in order to test if a generated track is challenging enough for a particular driver. Fitness metrics were used to evaluate the suitability of a new track for the controller. However, the research was focused on the methodology and creativity of the generated tracks instead of their evaluation with the real drivers.

Apart for just being entertaining and stimulating, games can be used for training and educating as well. Zyda (2005) refers to this as “Serious Games”; a medium to enhance education in a more entertaining way. Based on the notion of teaching through games, Backlund et al. (2006) conducted research on driver behaviour between people that play racing, action, sport games (RAS-gamers) and non-gamers. People were categorised into two groups through a questionnaire, whereas their driving skills (attention, decision making, risk assessment, etc.) and attitude (respect speed limits, speed margins and fellow drivers) were rated by driving school instructors, using 7-point Likert scale. Their findings show a positive correlation between gaming and skill oriented aspects of driving, although there was no statistically significant effect of attitude from the two groups. An important concluding statement suggested that games and more specifically driving simulators are able to provide positive effects on driving behaviour and user skill enhancement thus motivating further research.

## Methods and materials

Our approach of the user model implementation, described in Sect. 3.4, utilises a sequential hybrid modelling approach and integrates several algorithms, techniques and theories from literature. Figure 1 encapsulates the model processes and identifies the backbones of each layer. In this section we give an overall view of the methods adopted in our model and explain why and where we apply them.

### Theoretical background

In order to achieve high levels of engagement and training quality we employ ideas from educational theoretical frameworks such as *Concept of Flow*, *Zone of Proximal Development* and *Zone Theory*. The following section describes the various theoretical as well as psychological frameworks that inspired our user model implementation.

#### Trace Theory

As shown in Fig. 1, Trace Theory (TT) is responsible for bridging the low levels of our model to the higher ones through the transformation rules. TT is a popular and efficient framework for collecting, analysing and transforming users’ low level interactions, with a particular system, into more contextual and meaningful high-level traces. These low level interactions can be the inputs of the user to the system as mentioned by Clauzel et al. (2011). We define them as low level primitives. A formalisation of the trace framework found in Settouti et al. (2009) suggests that a particular combination of several low level primitives set by the system designer creates a primary trace.

As in Bouvier et al. (2014), primitives were set to be mouse and keyboard entries whereas a collection of them on specific timestamps and specific places in their user interface were generating the primary traces. However, a primary trace is still low level and not meaningful enough. The introduction of a rule based system that is determined by a system expert aims to transform the primary traces into higher level abstract entities that can remark on user’s profile and model. As a result of the rules being set by an expert, they are human explainable and therefore more specific about their underlying assumptions.

Our approach encapsulates the ideology of TT and makes use of the low level primitives, primary traces and transformed traces in order to create the model of the driver, while the player is operating the simulator. In Sect. 3.4.6 we provide a detailed description on how we adopt this framework into our model.

#### The concept of “flow”

The concept of Flow has been introduced by Csikszentmihalyi (1990), whereas being in Flow has been identified by Nakamura and Csikszentmihalyi (2002) as having certain perceptions. Flow represents the feeling of being completely immersed and engaged in an activity and also experiencing high levels of enjoyment and fulfilment (Csikszentmihalyi 1990). These kinds of activities were identified as self-motivating or “autotelic” and for an activity to be autotelic there has to be a balance between the challenge and the personal skill (Csikszentmihalyi 2000). In the case of challenge being higher than the skill then anxiety builds up whereas if the skill is higher than the challenge then boredom sets in. However, among different individuals the balance between those two entities are not of the same quantity.

Steels (2004) discusses the main components of an autotelic artificial intelligence agent that utilises the concept: (1) the user should feel in control and be able to alter the level challenge and (2) the agent should be capable of generating new experiences and challenges within the environment. Similarly, Chen (2007) mentioned the concept of Flow as a mechanism of keeping the game challenges and players skills in balance through the adaptation of game’s challenges in response to player’s actions.

Through our model, the aim is to create an autotelic activity by monitoring the skills and challenges of the user so as to adjust the difficulty of the game. As shown in Sect. 3.4.7, the main principles of the concept (Experience and Challenges) are evaluated through our model, at the highest level (see Fig. 1), to describe the current state of the user.

#### Zone of proximal development

Developmental research in children by  Vygotsky (1978) addressed the general relation between learning and development and the specific features of this relationship through the approach of “Zone of Proximal Development” (ZPD). In ZPD we determine at least two developmental levels. The first is the Actual Developmental Level (ADL), which defines the mental functions that have been established by the individual (completed the developmental cycles). The second is the Potential Development (PD), which is the development level that an individual can reach under guidance, assistance or collaboration. ZPD is the distance between ADL and PD and defines functions that are not yet matured but are in the process of maturation.

Demiris (2009) operationalised the ZPD concept through the creation of an hierarchical user model, which was used in order to infer the amount of assistance that should be given to a user driving a wheelchair.

In the racing experiments of this paper, each individual has different expertise and rate of learning, therefore a variety of training methods should be adapted to fit each of the users. In a task where the goal is similar (e.g. in a racing game: tackling a sharp turn with high speed), people with lower experience would have a larger ZPD and will need more assistance to reach the desired level. As shown in Sect. 4, through our data collection process we observed how the notion of ZPD operates on users with variant set of skills in driving. This was taken into account so that the model would create personalised decisions for each user.

#### Zone theory

Valsiner (1997)’s developed the *Zone Theory* to explain human development in educational setting, describing the process that establishes the development of a skill in an individual. Valsiner (1997)’s theoretical framework includes three zones:

- The Zone of Free Movement (ZFM) represents a cognitive structure of the relationship between a person and the environment in terms of constraints that limit the freedom of these actions and thoughts.
- The Zone of Promoted Action (ZPA)—defines the set of activities, objects or areas within the environment from which the tutor is promoting to the tutee. With the tutee having no obligation to accept it.
- The Zone of Proximal Development (ZPD) from Vygotsky (1978)—defines the amount of assistance the tutee needs to reach his/her potential development (PD).

These zones constitute an interdependent system between the constraints enforced on the environment of the learner and the actions being promoted for the learner where both constraints and actions are imposed by external stimulus.

The development is optimised when the relationship between ZPA and ZPD is such that what is promoted (ZPA) lies within the individual’s ability to achieve (ZPD). The ZPD cannot be fully incorporated in the ZFM. Since ZFM is determined by the tutor and a tutee can only develop aspects of the ZPD that have been advertised (ZPA) and not restricted (ZFM). When all zones merge it emerges “a family of possible novel forms of change” where ZPD is dependent on the state of the ZFM/ZPA complex (Galligan 2008).

In our case, the theory of zones serves as an interesting approach to explain the relationship between our users and our adaptive training framework. The ZFM describes the practical space that the user (tutee) can operate. This is the car simulator, the hardware utilised and the various tracks that are available. The ZPA represents the constraints that are promoted by the framework. The model tries to reach the engagement and learning goals set on development, and through the feedback that it receives from the user’s actions, it generates new tailored tracks that advertise new spaces (tracks) where learning and development are most likely to be realised for a particular user. An optimised relationship between ZPA and user’s ZPD is realised through the new track, however, the user has to willingly co-operate, with their engagement and attention, for the learning and development to take place, as suggested by Blanton et al. (2005) through the concept of *self-scaffolding* (Valsiner 2005).

### Methodological background

In this section we describe the algorithms adopted to perform feature extraction from the user’s data.

#### Affinity propagation

The first phase of the data collection process consists of storing streams of sequential data and clustering them into *Low Level Primitives* (Fig. 1). Ideally the clustering method should conform to the following requirements:

- Automatic determination of optimal number of clusters. The number of clusters is one of the extracted features of the user model.
- Flexibility on the choice of similarity measure to make the clusters more relative to the feature they describe.
- Simplicity and fast performance while maintaining low error levels. Our user modelling is performed in real-time.

As a result, the Affinity Propagation (AP) algorithm was chosen. AP (Frey and Dueck 2007) is an unsupervised clustering based approach. The method groups the data by finding data’s center points, called exemplars, through data specific similarity. The difference in approach with respect to other methods [e.g. k-centers (MacQueen et al. 1967)] is that the initial conditions suspect all data points as exemplars. Therefore, the number of clusters is not pre-defined but it can be influenced (if a prior information is known) by values in the similarity measure matrix. The novelty of the method is that it recursively transmits real-value messages throughout the data network of the suitability of each point being an exemplar, until a good set of clusters is found. In addition, the similarity measure matrix is a user defined function (usually Euclidean distance) whereas through its diagonal, the user can increase the values of some data points so that they are more likely to be used as exemplars. The diagonal is usually set to the median or the minimum of input similarities.

#### Random forests for real time head pose estimation

Apart from eye gaze, the other main physiological feature of our model is the real-time estimation of the head pose of the user. There are several reasons for combining eye gaze and head pose together. Our main considerations were to keep the costs low when providing sensors for our model and provide an unobtrusive experience. Our particular eye-tracker has a limited angle visibility. This means that it can no longer track the eyes of the user when head angles are large. Head position aids to fill in this gap of information when there are not any gaze data available.

Fanelli et al. (2011) used Random Forests (RFs) (Breiman 2001) to estimate, in real time, the 3D coordinates of the nose tip and the angles of rotation of the head through data obtained from a depth camera. In order to accomplish high prediction accuracy, they trained their algorithm using a generated large database of synthetic depth images of faces by rendering a 3D morphable model in different poses. Their method was proven to be fast and reliable even when there was a percentage of occlusion on the nose. This algorithm is suitable for our experiment since it can operate in real time, it does not need any manual initialisation to find the head pose and also does not require utilisation of GPU power like similar methods.

### Implementation

This section discusses the simulator used, the experimental protocol as well as the Framework’s configuration chosen for the data collection process, that aided in the design of our user model.

**Fig. 2:**
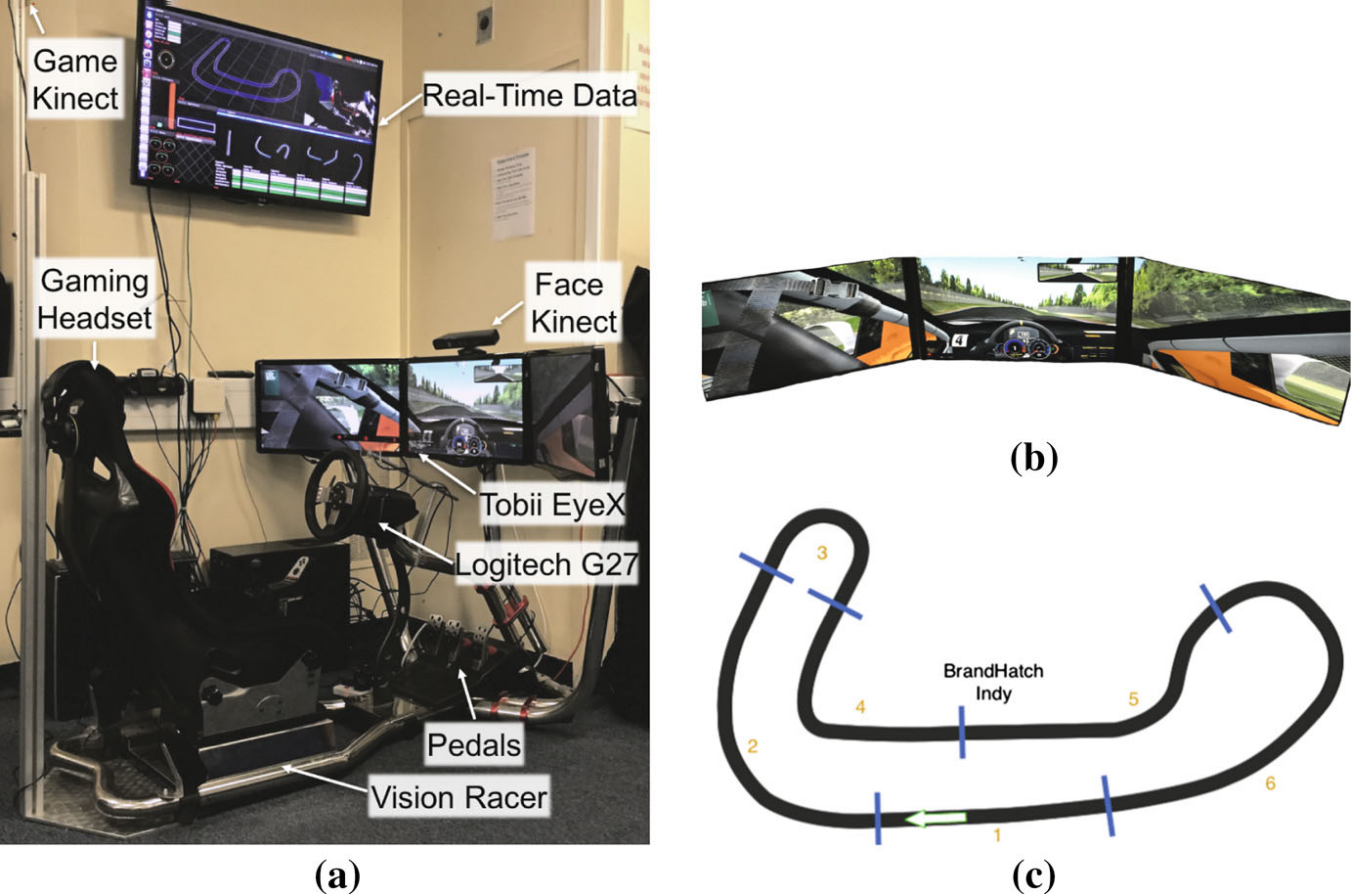
**a** Our custom car simulator setup consisting of Vision Racer Seat with a force feedback steering wheel, pedals and three monitors to enhance the user’s immersion to the game. Two RGB-D cameras as well as an eye tracker was installed to capture the user’s physiological signals. **b** Screenshot from our customised track. **c** Track used for the experiments split manually into six segments

#### The simulator

Data is collected through a racing car simulator software, rFactor,^1^ recommended for its excellent graphics and realistic vehicle dynamics by many users and racing teams. As shown in Fig. 2a, we are using a custom set-up of the Vision Racer VR3^2^ seat with Logitech G27 Force Feedback Steering Wheel and a combination of three monitors to enhance the user’s experience. We have installed a Tobii EyeX eye tracker and 2 RGB-D cameras (Kinect) which are positioned in such a way that one of them captures the player’s face and the other captures both the player’s actions and the output from the monitors. Real-time performance metrics, 3D-car position and user actions are being displayed on the wide screen monitor on the wall. For the experiment, the users were asked to complete 20 laps on a particular track that we developed and to fill a questionnaire with demographic and user profiling questions before and after the experiment.

#### Car and track selection

The car, selected from the ones available in the game, was a sport version of the Renault Megane. The requirements were to have a car that was easy to drive by an amateur driver, quite stable on the road, with a decent acceleration and top speed.

The experiments are loaded with a custom designed track called BrandHatch Indy, which is a short ($\approx$1.93 km) but still challenging track with various types of segments (e.g. chicanes, straights, sharp turns). As seen in Fig. 2c, the track was split into six segments which we use to group the data in the user model. Segments were defined in such a way that they will consist only of a single path type (e.g straight, turn, chicane, etc.). In the track’s implementation, each side of the road was fenced (after 20 m of grass), so the user cannot skip a segment by taking shortcuts.

#### Data collection

We collected data from **52 **users, where each user was asked to drive our customised track once for 20 laps. The user answered a set questions before and after the experiment. Prior to starting the experiment, each user was informed about the controls, the visual information of the simulator and the fastest lap of the particular track as a form of motivation.

It was important not to restrain the users with any unnecessary game-specific constraints or tasks. Thus users were advised to play and enjoy the game in their own way. This is because in a similar set up, Backlund et al. (2006) reported that users who were not assigned any task during a driving experiment enjoyed the game more than the ones who were given a task. Each subject was given the opportunity to get to know the simulator through a single practice lap at the beginning of the experiment which was discarded from further analysis. It is important to note that none of the users had previous experience on the particular game or track. At the end of the trial and after the completion of the questionnaire, the users were informed about the aims of the experiment and a short informal interview was carried out.

#### Questionnaire

Through questionnaires, we collected demographic as well as subjective metrics from our users. Subjective metrics were designed to aid the evaluation of the user model. As will be shown in Sect. 4, we based the validation of our user model’s outcomes and high level variables on the reports obtained by the users.

Before starting the experiment, the users were asked about their age, gender and if they had a driving license or not as well as how long they have been driving. Furthermore, they were asked questions (Likert-scale) shown in Table 1.

**Table 1:** Game-related questions asked before and after the experiment

ID	Questions	Options
Before the experiment		
*Frequency*	How often do you play car racing games?	[Never, Occasionally, Frequently, Every Day]
*pre Self-Rate*	How would you rate yourself in car racing games?	[Beginner, Intermediate, Advanced, Expert]
After the completion of the experiment		
*post Self-Rate*	How would you rate yourself in the racing game you tried?	[Beginner, Intermediate, Advanced, Expert]
*Difficulty*	How would you rate the path of the given track?	[Very Easy, Easy, Medium, Hard, Very Hard]
*Improved*	As you loop through the track did you feel that you improved?	[Nothing at all, Little Bit, Quite a lot, Very Much]
*Fatigue*	Did you feel tired during the experiment because of the lap repetition?	[Nothing at all, Little Bit, Quite a lot, Very Much]

### User modelling

Our approach to the user model implementation utilises a sequential hybrid modelling approach and integrates several algorithms, techniques and theories from literature. A top down view of the user model shown in Fig. 1 encapsulates the model processes and identifies the backbones of each layer.

Real time raw data from the user input, game, eye tracker and head pose (*Low Level Primitives*) are being converted into *Performance Metrics* by comparing them against the user’s previous “best” data or the expert’s data. Each metric has been given a certain weight through a linear model (*Weighting Model*), forecasting against the time taken for a certain path to be completed. Through the utilisation of *Trace Theory*, the performance primitives—along with their weights—are transformed via game specific rules, into variables of the concept of *flow* (Csikszentmihalyi 1990) (*Theoretical Frameworks*). This concept can evaluate the challenges that the user is facing (*Exploration*), the skills that the user attained (*Experience*) and the *Attention* that the user is paying to the task. From then on, the algorithm tries to balance those abstract concepts according to the individual by giving certain instructions to alter the track.

In this section we break down each of the layers of the user model—shown in Fig. 1—and explain the processes carried out to reach to a decision for each segment.

#### Low level primitives

Our focus was to use data from unobtrusive sensors, so that we can avoid the attachment of sensors to body parts, as this “might affect the experience of our user” (Yannakakis et al. 2013). Data from the game (e.g. position, speed, number of collisions), user’s actions, eye gaze and RGB-D data are collected during the task and timestamped through a common clock. One of the novelties of our method is that the data is grouped and analysed according to predefined split segments of the track (see Fig. 2c).

Segments can either be a turn, a straight line or a chicane. The advantage of this method is that the user is being monitored more frequently than once every lap and also our metrics reflect on the performance of more primitive paths where it is easier to identify the specific weaknesses of the driver. As seen in the bottom part of Fig. 1, the low level primitives of the user model consist of data from two main categories (*physiological* and *non-physiological* data), extensively listed in Table 2.

**Table 2:** The raw variables collected for the user model, directly from the sensors

Non-physiological		Physiological	
User inputs	Game outputs	Eye tracker	RGB-D
1. Braking	4. Time of collisions	10. Eye gaze (XY)	13. Camera information
2. Throttle	5. Car XYZ position	11. Eye position (XYZ)	14. Depth (m)
3. Steering (time)	6. Speed (km/h)	12. User presence (time)	15. RGB video (time)
	7. Virtual orientation		
	8. Lap No.		
	9. Time

**Table 3:** The primitives of the lowest level of the user model extracted from the raw variables

Non-physiological		Physiological	
User inputs	Game outputs	Eye tracker	Head pose
1. Average braking	6. No. of collisions	10. No. of blinks/s	17. CN of HP
2. Average throttle	7. Car XYZ position	11. Screen concentration	18. CC of HP
3. Average steering	8. Position and speed	12. CN of EG	19. CN of HP and VO
4. CN of user inputs	9. (Segment time)	13. CC of EG	20. CC of HP and VO
5. CC of user inputs		14. CN of EG and VO	
		15. CC of EG and VO	
		16. Eye fixations	

*CN* cluster number, *CC* cluster centres, *VO* virtual orientation, *EG* eye gaze, *HP* head pose

#### Analysis of low primitives

The raw data of Table 2 cannot be directly used as a comparison measure for creating a performance metric. It has to be transformed into a value or sequence of values that can exert a significant performance result when contrasted with ones of the same kind. Over the next paragraphs we are going to describe the process of converting the variables of Table 2 into the primitives of Table 3.

*User inputs* These are the principal links between the game and the user. The user has three main inputs to the car simulator: the steering wheel, the throttle and the brake pedals. For each of the three series of data we compute the mean value for a particular path to create the corresponding primitives.

We also perform the Affinity Propagation (AP) clustering method, described in Sect. 3.2.1 over all three inputs to find the number and position of their dominant values along the path. Clustering results point to several combinations of inputs that act as the centres of all data. The number and values of these centres are both used as a primitive in the user model. AP clustering facilitates the use of a user specified similarity measure to group the data into clusters. The similarity measure used to cluster the three inputs is their Euclidean distance.

*Game outputs* The simulator we are using provides real time output data of the car and the environment’s states with a high sampling rate (100 Hz). By using the lap number, time and path position we are able to create indices at the points at which each segment starts and ends for each user, so we can split and group the data together. As game output primitives, we extract the number of collisions per second for each segment. We also store primitives of the user’s path as well as the user’s path accompanied with the car speed.

*Eye tracker* From the eye tracker we obtain four kinds of information; a parameterised real world position of the eyes, the eye gaze on the screen, the eye gaze fixations and the presence of a user. Through a combination of this information, we calculate and define as the user’s primitives (see Table 3): the number of blinks, the number of fixations per second for a particular segment, as well as the screen concentration time normalised to the segment’s time.

We also perform AP clustering on the eye gaze to determine the position and the number of important targets on which the user was fixating. Instead of clustering only on the eye gaze, we determine the number and important centres of user’s gaze according to the orientation of the car in the virtual space for a particular segment. This addition encapsulates the relationship between the user’s gaze and the orientation (in radians) of the virtual world in the game. The similarity measure used for both clustering approaches is the Euclidean distance of the 2 and 3 dimensions respectively.

*RGB-D and head pose* Apart from using the cameras to record the experiments, we also use the depth information to find the head pose of the user. Incorporating the algorithm, described in Sect. 3.2.2, we determine the head position and orientation of the user at any given time. As with the eye gaze, we use the orientation data as well as the virtual orientation of the game to create two sets of clusters with the AP algorithm. This results in four different primitives regarding the head pose: number of spots and the location of the centres for each of the approaches. The orientation of the head pose is given as a quaternion. Therefore, for a similarity measure in the AP algorithm we use a dot product to relate the angles between different quaternions. In addition, in the approach with the added virtual orientation, the extra dimension is related with the Euclidean distance.

After the primitives are computed, they are stored into the lowest level of the user model. The next step is to convert them into *performance metrics* so that we can assess the efforts of the user towards the task.

#### Performance metrics

An interesting study by Hong and Liu (2003) analysed the thinking strategies between different users in a game of “Klotski”.^3^ They concluded that expert users, determined by the fewer number of steps and operation time, were using more analogical thinking towards a solution (e.g. devising a series of next moves) whereas novice players’ movements were mostly random. Having in mind that the solving ability for a problem is content oriented, they suggested that by understanding the ways the experts operate, we can determine the type of training required for a novice learner to acquire the experts’ skills faster. The concept of our performance metrics employ this idea by assessing the user’s actions with the expert’s.

In the user model structure (see Fig. 1), the layer above the *low level primitives* involves their conversion to performance metrics. From each category in Table 3 several performance metrics are obtained comparing either the current user’s values to an “expert driver” (if they exist) or to a previous user “best”. Therefore, there are metrics appraising the performance of the user at a global level and at a personal level.

A collection of data for a segment is defined to be more optimal than others according to the amount of time the segment took to complete. As a result, we would expect that the “expert driver” would have the fastest possible time on that segment. In our experiments, the expert driver for each segment was defined at the end of the data collection process by selecting the user with the fastest segment times.

For each of the primitives in Table 3 we use different methods as comparison against the “optimal” values. Most of the primitives (Nos. 1–4, 6, 10–12, 14, 16, 17, 19) create the metric by finding the absolute difference from the optimal values, however, *time performance* (No. 9) is being divided by the optimal one for each segment. *Cluster centres* (Nos. 5, 13, 15, 18, 20) are being matched with their closest optimal centre and the average value of their Euclidean distance is obtained.

For Nos. 7 and 8 we use a different approach, since they consist of a sequence of data. In general, to find the dissimilarity of the path covered between two different sequences of car positions, it would be wrong to compare the points one-to-one since there is a high probability that the sequences are not of the same size (since the path has a very low probability of being identical but the sampling rate is constant). A solution is to use a form of dynamic time warping between the two sequences so that the temporal domain is abstracted out and we are only comparing the spatial domain which is the path. In our case, we simply find the two closest points of the optimal path to each of the user’s points in the path. We then calculate the point that is perpendicular to the vector joining the two optimal points and the user’s point.

**Fig. 3:**
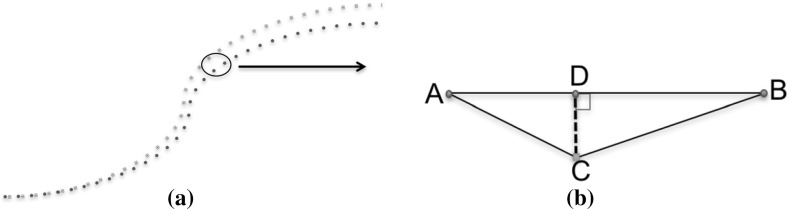
**a** Comparison of optimal (*blue*—*circles*) and user’s (*red*—*squares*) path. **b** Optimal points (*blue*—*circles*) and user point (*red*—*square*) forming the vectors. (Color figure online)

For example, Fig. 3a shows, in blue, the trajectory of the *expert* driver and in red is the recorded user’s trajectory. If we zoom in on the points, as shown in Fig. 3b, we have *A* and *B*, which are two consecutive points of the optimal path and *C*. *C* is a point created by the path of the user in the track. Our algorithm tries to find the closest points from the optimal path, *A* and *B*, for every user point, *C*. It then finds a point *D* on the vector  that forms a perpendicular vector . This calculates the difference of point *C* to its closest point on the optimal path. However, the path consists of discrete points and therefore, a perpendicular point on the path might not exist. *D* is found by optimising the solution for the 3 dot product equations shown in (1). The summation of all the absolute distances of the points in the user’s path, denoted by the vector  creates the *path performance* metric (No. 7 in Table 3) of the user.1

For calculating the *path with speed* metric (No. 8 in Table 3) we use a similar method as with the path metric. This time we have to find the speed of the new Point *D* as well. Therefore, speed is found by interpolating the speeds of points *A* and *B* using their distances as a weight as shown by (2). Then the sum of their squared differences (SSD) of all matched speeds is noted as a metric, as shown by (3).

2 $$\begin{aligned} {\textit{Speed}}_D= & {} {\textit{Speed}}_A + ({\textit{Speed}}_B - {\textit{Speed}}_A) * \frac{||AD ||}{||AB ||} \end{aligned}$$

3 $$\begin{aligned} {\textit{Metric}}_8= & {} \sum {({\textit{UserSpeed}} - {\textit{Speed}}_D) ^ 2} \end{aligned}$$

The presumption made to create those two metrics is that the former analyses the user performance only on the closeness to the optimal path, whereas the latter comments on the speed as well. As we notice later in Sect. 3.4.4, the latter is more correlated to the segment time since time is indirectly involved in the speed-path points.

The performance metrics in the model are converted to represent percentages, through exponential functions, so that (1) they are comparable against each other, without the need of rescaling, and also (2) outliers are brought closer together. Regarding the latter, when two users perform poorly in a metric, their values are expected to be well beyond and below the mean of an average user. However, the two values could still be far from each other. This doesn’t provide any more information rather than a poor performance from both on the particular metric.

The use of exponential functions helps to decrease that gap and keep the linearity of the rest, up to the point where the original value is significant. For example, if two users’ paths have a huge difference from the expert’s then we know that both users’ time and path performance would be low. However, the time versus path performance proportionality is not as linear as the ones coming from average-high metric performances.

The exponential function used to convert the metrics to percentages is shown in (4). Each performance metric is using a modified exponential function according to the metric value, *X*, of the expert to expert comparison. The Constant, *C*, for each different metric is determined by solving (4). This is by assigning the *Metric* variable to the median value of all the metrics collected (from all users for the particular metric), and the %*Metric* variable to 50%.

4 $$\begin{aligned} \%{\textit{Metric}} = 100*\exp \Big (-\frac{({\textit{Metric}}-X)}{C}\Big ) \quad \quad \forall \mathbf {R}~\text {and} X= \text {Expert-To-Expert value} \end{aligned}$$

#### Performance metrics weights

In racing, “time” is the most general and important metric that can reflect on the performance, skills and engagement of the user to the task. However, we have to get deeper into the data in order to understand whether the driver actually achieved a good time. In our user model we try to justify and break down the time taken by the user, through a particular segment, using several descriptive metrics. Therefore for finding the best “weight” of each metric towards the user’s segment time performance, we used various modelling approaches; whereas the best weights describing the correlation (5) from our collected data were adopted.

The metrics are split into three groups: All, Non-Physio, Physio. *All* includes all metrics in Table 3, *Non-Physio* consists of all metrics obtained from the user inputs and game outputs and *Physio* includes metrics associated with the user’s physiological data, such as those obtained from eye tracker and head pose. Time performance is not included in any groups since it is used as reference variable in (5).

5 $$\begin{aligned} \rho _{{\textit{max}}} = \rho \left( {\textit{TimeMetric}}_{s}^l, \sum _{k=1}^{m} W_{k}^s M_{k}^{sl}\right) \end{aligned}$$

where

- $\rho$ is the correlation coefficient
- ${\textit{TimeMetric}}_{s}^l$ is the time performance metric of a segment *s* and user lap *l* (No. 9 in Table 3)
- $W^s_k$ is the weight for a particular segment *s* of a particular metric *k*
- *m* is the number of metrics defined in each of their group: All, Non-Physio, Physio
- $M^{sl}_k$ is the Performance Metric *k* of segment *s* and lap *l*.

**Table 4:** Spearman correlation values of the performance metrics against segment time between user and expert data

Metrics	Rho-value (user)	Rho-value (expert)
1. Cluster number of eye gaze and virtual orientation	0.37	0.35
2. Cluster centres of eye gaze and virtual orientation	**0.41**	0.28
3. Cluster number of eye gaze	0.27	0.19
4. Cluster centres of eye gaze	0.31	0.14
5. No. of blinks/s	0.27	0.17
6. Screen time	0.28	0.17
7. No. of collisions/s	**0.56**	**0.49**
8. Path with speed performance	**0.82**	**0.91**
9. Path performance	**0.57**	**0.48**
10. Cluster number of head pose and virtual orientation	**0.44**	**0.49**
11. Cluster centres of head pose and virtual orientation	**0.50**	0.38
12. Cluster number of head pose	0.23	0.14
13. Cluster centres of head pose	**0.40**	0.11
14. Cluster centres of user inputs	**0.59**	**0.44**
15. Cluster number of user inputs	**0.49**	**0.54**
16. Average braking	0.21	0.25
17. Average throttle	**0.50**	**0.60**
18. Average steering	**0.48**	0.33
19. Eye Fixations	0.20	0.17

All *p* values are ${<}0.001$. The closer the value is to 1 the closer the particular variable is more correlated to the segment time. As anticipated the physiological signals have on average lower significance to the time than the non-physiological. (Values ${\ge }0.40$ are in bold)

**Table 5:** Spearman correlation values of the performance metrics against each segment time between user and expert data

	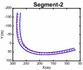		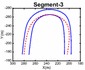		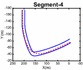		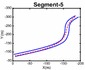		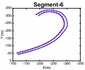	
Metrics	Rho (user)	Rho (expert)	Rho (user)	Rho (expert)	Rho (user)	Rho (expert)	Rho (user)	Rho (expert)	Rho (user)	Rho (expert)
1. CN of EG and VO	0.32	0.36	0.37	0.28	**0.42**	0.34	0.25	0.27	**0.40**	**0.43**
2. CC of EG and VO	0.39	0.33	**0.45**	0.23	**0.43**	**0.40**	0.30	0.24	**0.51**	0.37
3. CN of EG	0.22	0.24	0.25	0.20	0.27	0.29	0.19	0.13	0.38	0.08
4. CC of EG	0.33	0.18	0.32	0.20	0.31	0.16	0.21	0.19	0.39	0.21
5. No. of blinks/s	0.23	0.18	0.24	0.04	0.28	0.22	0.30	0.23	0.29	0.23
6. Screen time	0.27	0.23	0.28	0.01	0.27	0.25	0.30	0.26	0.31	0.15
7. No. of collisions/s	**0.50**	**0.49**	**0.60**	**0.60**	**0.55**	**0.50**	0.25	0.23	**0.69**	**0.58**
8. Path with speed Perf.	**0.82**	**0.98**	**0.86**	**0.92**	**0.91**	**0.98**	**0.89**	**0.99**	**0.84**	**0.93**
9. Path performance	**0.45**	0.34	**0.66**	**0.56**	**0.48**	0.35	0.35	0.33	**0.75**	**0.68**
10. CN of HP and VO	**0.48**	**0.54**	0.36	0.33	**0.49**	**0.53**	0.30	**0.42**	**0.51**	**0.61**
11. CC of HP and VO	**0.55**	**0.43**	**0.44**	**0.42**	**0.54**	**0.44**	0.38	0.35	**0.63**	**0.45**
12. CN of HP	0.26	0.31	0.27	0.29	0.19	0.23	0.06	0.04	0.31	0.36
13. CC of HP	0.37	0.09	0.36	0.09	0.37	0.07	0.27	0.11	**0.56**	0.19
14. CC of user inputs	**0.53**	**0.40**	**0.64**	**0.60**	**0.58**	**0.43**	0.31	0.04	**0.76**	**0.64**
15. CN of user inputs	**0.44**	**0.41**	**0.49**	**0.45**	**0.51**	**0.67**	0.27	**0.43**	**0.62**	**0.61**
16. Average braking	0.18	**0.45**	0.35	**0.45**	0.19	0.20	0.15	**0.46**	0.26	0.34
17. Average throttle	**0.40**	**0.60**	**0.43**	**0.51**	**0.58**	**0.73**	0.36	**0.54**	**0.69**	**0.72**
18. Average steering	**0.40**	0.25	**0.46**	**0.47**	**0.43**	0.33	0.32	0.16	**0.64**	**0.44**
19. Eye fixations	0.20	0.22	0.18	0.23	0.20	0.02	0.15	0.11	0.25	0.23

Images show the path of each segment along with the expert’s trial. All *p* values are ${<}0.001$ and therefore correlations are statistically significant. Correlations which are close to 0 show that the values obtained from the particular metric didn’t reveal any relationship to the improvement of segment time. (Values ${\ge } 0.40$ are in bold)

*CN* cluster number, *CC* cluster centres, *VO* virtual orientation, *EG* eye gaze, *HP* head pose

We evaluated the weights through various models, using different versions of Spearman correlation and linear regression:

- *Spearman correlation* The first approach to determine the relation and significance of each metric to time is to perform non-parametric Spearman’s correlation (Spearman 1904) between each performance metric and their respective performance segment time. The statistical significance of the correlation values is defined by the *p* values ($p <0.05$).
  The correlation results are shown in Table 4. As previously mentioned, metrics are either created from comparisons with the *expert’s* or the *user’s* “*best*” data. All metrics’ *p* values are well below 0.001, indicating that the results are statistically significant. It is also noticeable that most of the correlations coming from the comparisons of *user’s* “*best*” metrics are larger than those of the *expert*. This is expected since each user will behave according to their own knowledge and skills, therefore a small change towards improvement will be more noticeable. Evaluating (5) , the weights $W^s_k$ are set to be the normalised value of the correlations found from each metric *k*. Also, since the Spearman correlation is performed from all data collected including all segments, the weights of each segment *s* are the same.
- *Spearman segment correlation* By grouping the data according to their segments, we performed Spearman correlation within each segment. This approach revealed that the significance of each performance metric also depends on the segment’s path as well (see Table 5). Therefore, for this second method we set the weights $W^s_k$ to be the normalised correlations found for each individual segment *s*. Thus, the difference of this method to the previous is that the weight of each metric *k* is varying across the segments. Segment 1, which is the straight line in the path, was not included in the model since it has to remain unaltered due to track design issues.
- *Linear regression (LR)* The main issue with both of the previous approaches is that the Spearman correlation calculates the significance of each metric without considering the correlations between them as well. If two metrics are highly correlated to each other then one of them can be removed since this makes the whole model simpler and more robust to small changes. In order to reduce those redundancies and find the best weights that describe (5), we perform a linear regression for each group of segment data, according to (6).
  6 $$\begin{aligned} TimeMetric_{s}^l = C + \sum _{k=1}^{m} X_{k}^s M_{k}^{sl} \end{aligned}$$

The linear regression model calculates the coefficients $X_{k}^s$ of each metric *k* for a particular segment *s* accompanied with an interception coefficient *C*. Coefficients are found using the least squares problem method by minimising the sum of the squared error ($\min \left||Ax - b\right||_{2}^{2}$) where in our case *A* represents a matrix of our metrics observations and *b* is their corresponding time metrics (Mead and Renaut 2010; Lawson and Hanson 1974). We experimented with six different approaches using this model (The name in the brackets specifies the id given to each of the approaches):

1. (*LM Perc*) The linear model was forced to provide either positive or zero valued coefficients $X_{k}^s$ (*C* was unconstrained) for solving (6), where the data were all expressed as percentages. Then the coefficients for each segment $X_{k}^s$ were normalised and set as the weights $W^s_k$. The reasoning for the positive bounds is because metrics were designed to have positive correlations to time and also the conversion of the metrics to percentages was one sided with 100% being the expert.
2. (*LM Pos Raw*) The linear model was bound to calculate positive coefficients but metrics were not converted into percentages. The coefficients found for each segment $X_{k}^s$ were then set directly as the weights $W^s_k$.
3. (*LM Raw*) In order to check that our reasoning for positive bounds was valid, we used the same approach as 2 but with no positive bounds. Therefore, coefficients could be negative as well.
4. (LM * OPT) All of the above linear models are set with the maximum number of metrics available. However, this might impact on fitness of the model since they could provide coefficients that are overfitting the data due to the high complexity of the model and the degrees of freedom introduced. Therefore, using all of the three methods mentioned (*LM Perc, LM Pos Raw, LM Raw*), we found the optimal combination and number of metrics of each method that gave the best linear model by finding the lowest calculated root mean square error (RMSE)—all combinations were tested using exhaustive search.

#### Weight model selection

**Fig. 4:**
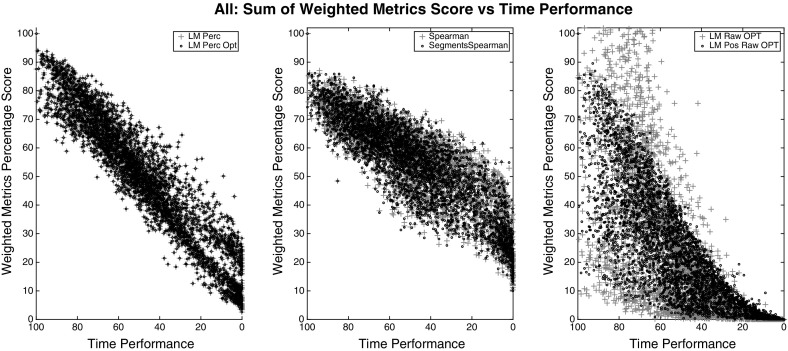
Outcomes of different models for group *All*. Between the models tested, *LM Perc* provides the best weights for a linear correlation between the combined metrics and the time performance. *LM Perc* and *LM Perc Opt* have identical correlations ($\rho = 0.95$) since the latter only corrected some peaks in the values

**Fig. 5:**
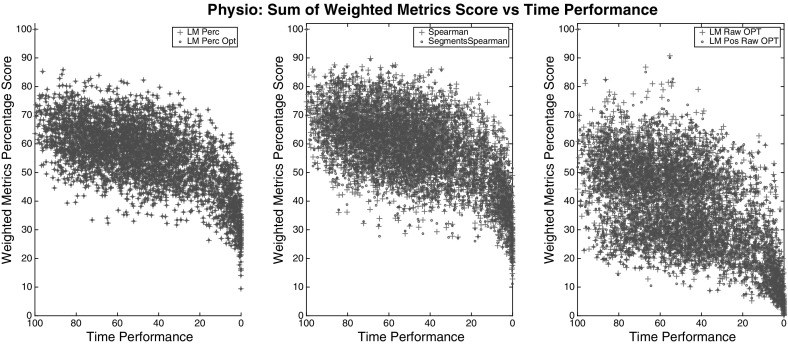
Outcomes of different models for group *Physio*. Between the models tested, *LM Perc* provides the best weights for a linear correlation between the combined physio metrics and the time performance. *LM Perc* and *LM Perc Opt* have identical correlations ($\rho = 0.58$) since the latter only corrected some peaks in the values

**Fig. 6:**
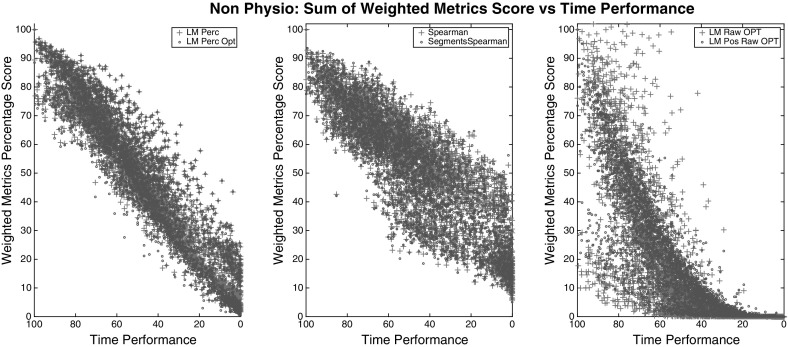
Outcomes of different models for group *Non-Physio*. Between the models tested, *LM Perc* provides the best weights for a linear correlation between the combined non-physio metrics and the time performance. *LM Perc* and *LM Perc Opt* have identical correlations ($\rho = 0.94$) since the latter only corrected some peaks in the values

For choosing the right weights that fit the data into (5), we evaluated the users’ data from the weights obtained from each model and then performed a Spearman correlation test between the *Time Metric* (right side) and the summation of the left side of (5), for each group. The Spearman correlation of all models and groups are shown in Table 6 whereas the plots in Figs. 4, 5, and 6 show only the best of each model type for clarity.

The most correlated model, among all groups of metrics, was the *LM Perc* where the weights were bound to be positive and data were converted to percentages. All versions of the optimal *LM* models (LM * Opt) didn’t substantially improve their correlation result but through the plots we can spot that the optimal models corrected some peaks in the values. Each dot in the figures represents a segment trial from a particular user. Therefore, each dot is a set of metrics collected from a respective segment (multiplied by their respective weights), plotted against the time metric that is normalised so we can compare all the data from all segments together.

The model selected for our *Framework* is the LM Percentage (*LM Perc*) since it gives the highest Spearman correlation and also the steeper linear curve in all the results. The weights used for each metric generated from expert comparisons are shown in Tables 7, 8, and 9. The metrics that haven’t been found to be significant in the user model or they are already described by other metrics, have a very low weight value.

**Table 6:** Spearman correlation of the model’s outcomes for three different group of variables; *All*, *Physio*, *Non-Physio*

Figure ID	Metrics	All	Physio	Non physio
*LM Perc*	1. LM percentage	0.95	0.58	0.95
*LM Perc Opt*	2. LM percentage optimal	0.95	0.58	0.94
*Spearman*	3. Spearman	0.86	0.54	0.86
*SegmentsSpearman*	4. Spearman segments	0.87	0.56	0.87
	5. LM raw	0.50	0.58	0.55
*LM Raw Opt*	6. LM raw optimal	0.73	0.58	0.92
	7. LM raw positive	0.86	0.56	0.92
*LM Pos Raw Opt*	8. LM raw positive optimal	0.87	0.56	0.92

All correlations are statistically significant with *p* values ${<}0.01$

#### Transformation rules

The metric-weight pairs described in the previous sections specify the significance of each metric to the good time performance of a particular path. They are the fundamental information that transformation rules are using to define the higher level concepts.

Following the theoretical frameworks of behavioural analysis like the Concept of Flow (Steels 2004; Csikszentmihalyi 1990), the Zone of Proximal Development (Vygotsky 1978), the Zone Theory (Valsiner 1997) and the Trace-Based System theory described in Settouti et al. (2009) and Bouvier et al. (2014), we further analysed the metrics using game specific assumptions into three classes that make up the high level in the driver model (called *Transformed Modelled Traces* in Trace Theory): *Experience*, *Exploration* and *Attention* The value of each class is determined by a combination of the performance metrics analysed according to certain game-specific rules:

**Table 7:** Weights of each performance metric in the *All* group, generated from the expert comparisons using the linear regression model constrained for positive value coefficients (*LM Perc*)

Metrics	Segment 2	Segment 3	Segment 4	Segment 5	Segment 6
1. CN of EG and VO	**0.020**	**0.004**	**0.007**	**0.008**	**0.010**
2. CC of EG and VO	**0.027**	**0.051**	2.2e$-$06	**0.024**	**0.080**
3. CN of EG	1.8e$-$11	**0.014**	5.8e$-$08	**0.004**	**0.002**
4. CC of EG	7.3e$-$10	**0.016**	**0.025**	3.4e$-$10	3.3e$-$11
5. No. of blinks/s	**0.011**	4.1e$-$10	3.4e$-$10	4.1e$-$10	**0.014**
6. Screen time	2.2e$-$11	4.8e$-$10	**0.017**	**0.005**	4.9e$-$12
7. No. of collisions/s	1.6e$-$10	2.0e$-$11	6.3e$-$09	**0.042**	**0.018**
8. Path with speed Perf.	**0.674**	**0.467**	**0.848**	**0.768**	**0.423**
9. Path performance	6.0e$-$12	3.5e$-$11	2.0e$-$10	**0.011**	1.6e$-$12
10. CN of HP and VO	**0.016**	**0.004**	**0.014**	**0.015**	**0.015**
11. CC of HP and VO	**0.039**	**0.097**	**0.002**	1.4e$-$10	**0.058**
12. CN of HP	**0.013**	4.3e$-$10	6.2e$-$08	**0.008**	**0.018**
13. CC of HP	**0.008**	2.5e$-$12	**0.005**	**0.033**	2.4e$-$11
14. CC of user inputs	**0.027**	**0.044**	3.5e$-$08	7.2e$-$07	**0.048**
15. CN of user inputs	**0.021**	**0.047**	**0.008**	**0.012**	**0.015**
16. Average braking	2.6e$-$11	**0.038**	1.1e$-$07	**0.009**	**0.050**
17. Average throttle	**0.124**	**0.151**	**0.075**	**0.047**	**0.244**
18. Average steering	**0.014**	**0.026**	2.4e$-$08	**0.014**	3.2e$-$12
19. Eye fixations	**0.004**	**0.042**	8.8e$-$07	1.2e$-$10	**0.006**

Table shows the amount of significance of a metric according to the segment’s path. Each column adds up to one. These values were used to derive our results in the *expert* model

*CN* cluster number, *CC* cluster centres, *VO* virtual orientation, *EG* eye gaze, *HP* head pose

**Table 8:** Weights of each performance metric in the *Physio* group, generated from the expert comparisons using the linear regression model constrained for positive value coefficients (*LM Perc*)

Metrics	Segment 2	Segment 3	Segment 4	Segment 5	Segment 6
1. CN of EG and VO	**0.084**	**0.057**	**0.036**	**0.102**	**0.125**
2. CC of EG and VO	**0.216**	**0.088**	**0.335**	**0.218**	**0.214**
3. CN of EG	**0.049**	**0.063**	**0.056**	2.7e$-$07	**0.020**
4. CC of EG	1.5e$-$08	**0.048**	1.5e$-$06	1.2e$-$07	9.6e$-$09
5. No. of blinks/s	**0.074**	8.0e$-$09	**0.017**	**0.106**	**0.067**
6. Screen time	2.3e$-$06	6.6e$-$10	**0.038**	2.0e$-$06	2.6e$-$09
7. CN of HP and VO	**0.154**	**0.125**	**0.159**	**0.189**	**0.191**
8. CC of HP and VO	**0.303**	**0.367**	**0.319**	**0.385**	**0.148**
9. CN of HP	**0.062**	**0.170**	**0.041**	5.0e$-$08	**0.105**
10. CC of HP	2.1e$-$08	6.0e$-$10	3.2e$-$08	3.8e$-$08	**0.084**
11. Eye fixations	**0.057**	**0.081**	2.6e$-$08	1.4e$-$08	**0.045**

Table shows the amount of significance of a metric according to the segment’s path. Each column adds up to one. These values were used to derive our results in the *expert* model

*CN* cluster number, *CC* cluster centres, *VO* virtual orientation, *EG* eye gaze, *HP* head pose

**Table 9:** Weights of each performance metric in the *Non physio* group, generated from the expert comparisons using the linear regression model constrained for positive value coefficients (*LM Perc*)

Metrics	Segment 2	Segment 3	Segment 4	Segment 5	Segment 6
1. Path with speed Perf.	**0.776**	**0.611**	**0.914**	**0.842**	**0.545**
2. Path performance	1.3e$-$06	3.1e$-$07	7.0e$-$11	**0.011**	4.0e$-$07
3. CC of user inputs	**0.040**	**0.042**	1.3e$-$09	6.5e$-$09	**0.053**
4. CN of user inputs	**0.031**	**0.070**	**0.014**	**0.025**	**0.029**
5. Average braking	1.1e$-$06	**0.065**	1.6e$-$11	**0.013**	**0.063**
6. Average throttle	**0.133**	**0.178**	**0.073**	**0.039**	**0.288**
7. Average steering	**0.020**	**0.036**	1.1e$-$10	**0.020**	1.9e$-$07
8. No. of collisions/s	6.5e$-$09	8.6e$-$07	6.4e$-$12	**0.050**	**0.021**

Table shows the amount of significance of a metric according to the segment’s path. Each column adds up to one. These values were used to derive our results in the *expert* model

*Experience* The skills of the user, is determined by the proximity of the user’s primitives to the *expert*’s. Since the metrics are now expressed as percentages, the higher the value the better the user is performing on that particular metric. Although the significance of this metric to the user’s skill is determined by the weight calculated using the chosen linear regression method (*LM Perc*). Skill cannot be determined by a single group of values. It has to be an overall value of several trials. Therefore, it is calculated by the weighted sum of the mean of each performance metric over a certain number of laps, as shown by (7). It can be argued that *Experience* could also be determined by the average lap time of the user, as (7) is similar to (6). However, with (7) we are able to comment on the specific aspects (through the weight-metric pairs) that derive a high/low value. This feature broadens the use of our Framework, as we will discuss later in Sect. 5.1.

7 $$\begin{aligned} {\textit{Experience}}_{n}^{l,s} = \sum _{k=1}^{m} W_k^s * \frac{\sum _{i=l+(1-n)}^{l} M_{k}^{i,s}}{n}, \quad l>n, \quad n>0, \quad s \in S \end{aligned}$$

where

- *s* is the id of a particular segment of segment’s set *S*
- *l* is the lap number we are interested in
- *n* defines the number of laps the value is defined from
- $W_{k}^s$ is the normalised weight of a metric *k* and segment *s* in the *All* group
- *m* is the number of all metrics defined in the *All* group
- $M^{i,s}_k$ is the Performance Metric *k* of lap *i* and segment *s*
- $Experience_{n}^{l,s}$ is the *experience* value of lap number *l* and segment *s* over *n* laps.

*Exploration* As defined in the introduction, is a metric of how varied (explorative) is the range of actions that the user is using in the current task. Through the metrics we define this as the weighted sum, of a metric’s absolute shift to a proportion of its mean (defined as “jump”). If the difference between two consecutive metrics passes a fixed percentage value of the current experience then the value is positive, otherwise it is negative. This is expressed by (8).

8 $$\begin{aligned} Exploration_{n}^{l,s}= & {} \sum _{k=1}^{m} W_{k}^s * \Big (\big (| M_{k}^{l,s} - M_{k}^{l-1,s}|\big ) - \big (J^s_k*\frac{\sum _{i=l+(1-n)}^{l} M_{k}^{i,s}}{n} \big )\Big ), \nonumber \\&1>J_k>0,\quad l>n, \quad n>0, \quad l>1, \quad s \in S \end{aligned}$$

where

- $J^s_k$ defines the proportion of “jump” of the *experience* value of that metric
- $Exploration_{n}^{l,s}$ is the *exploration* value of lap number *l* and segment *s* from which current *experience* value for each metric *k* was calculated over *n* laps.

*Attention* keeps a record of the continuous attention of the user along consecutive segments. This is calculated by first evaluating the *experience* of the user only from *non-physiological* data through consecutive segments. If the value is above a threshold, then the user’s attention is high and positive and the value is kept as the *Attention*. Otherwise, we calculate the *exploration* value only from the *physiological* data and use that for *Attention*. This is expressed by (9).

9 $$\begin{aligned} \begin{aligned} I&= f(l,s), \\ {}^{np}{\textit{Experience}}_{n}^{l,s}&= \sum _{k=1}^{{np}_m} {}^{np}W_k^s * \frac{\sum _{i=I+(1-n)}^{I} M_{k}^{i}}{n}, \\ {}^{p}{\textit{Exploration}}_{n}^{l,s}&= \sum _{k=1}^{{p}_m} {}^{p}W_{k}^s * \Big (\big ( |M_{k}^{I} - M_{k}^{I-1}|\big ) - \big (^{p}J_k^s *\frac{\sum _{i=I+(1-n)}^{I} M_{k}^{i}}{n} \big )\Big ), \\ Attention_{n}^{l,s}&= {\left\{ \begin{array}{ll} {}^{np}{\textit{Experience}}_{n}^{l,s} - {^{np}T} &{}\text {if }\;{}^{np}{\textit{Experience}}_{n}^{l,s} \ge {^{np}T}\\ {}^{p}{\textit{Exploration}}_{n}^{l,s} &{}\text {if }\;{}^{np}Experience_{n}^{l,s} < {^{np}T}\\ \end{array}\right. }\\&\quad 1>{^{p}J_k^s}>0, \quad l>n, \quad n>0, \quad l>1, \quad s \in S \end{aligned} \end{aligned}$$

where

- *np* and *p* prescripts define the *Non-Physio* and the *Physio* group metrics respectively
- *I* is the index number representing segment *s* in lap *l*. Function *f* transforms *s* and *l* to I so that $I-1$ defines the previous consecutive segment of index *I*
- *n* defines the number of consecutive segments the value is defined from
- $W_k$ is the normalised weight of a metric *k* defined in the respective group
- *m* is the number of metrics defined in each group
- $M^{i}_k$ is the Performance Metric *k* of segment-lap index *i*
- $J_k$ defines the proportion of “*jump*” of the Experience value of that metric
- $^{np}T$ defines the threshold set for separating low and high values in the *Non-Physio* group *np*.

To sum up, we described the way the performance metrics are created, how their weights are evaluated and defined the values of our high level variables through game specific rules. The next step is to utilise these values to determine the current state and performance of the user and provide instructions to change the segments of the track.

#### Exploiting the flow

**Fig. 7:**
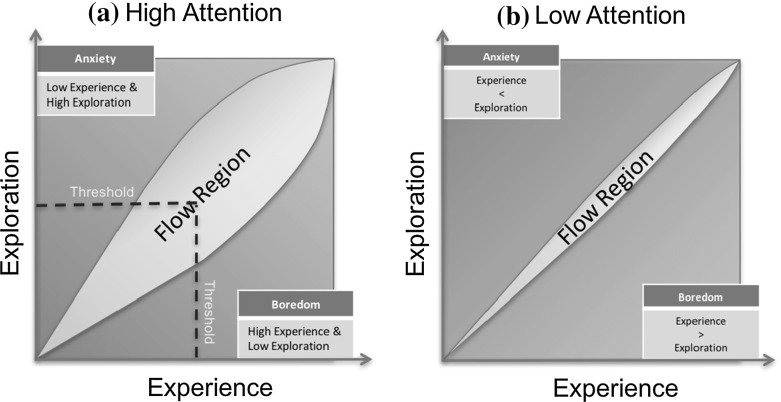
The two figures show how the area of the region of *flow* reduces between high and low *Attention*. The *flow* region defines the optimal region for user engagement. In the low *attention* case, the region of *flow* becomes narrower and the user model’s variables are compared between themselves than to the threshold parameters

The high level variables mentioned in Sect. 3.4.6 monitor the user experience while playing the game and control the *segment altering decision* algorithm according to the combination of their values. The balance between the level of challenge and the user’s skill is considered as the optimal operating region (*Flow Region*) whereas attention defines the range they are operating in, as shown by Fig. 7. When *attention* is low then the *flow region* is much narrower, therefore, *experience* and *exploration* values should be treated with more care than when *attention* is high. Our approach is that thresholds define low/high *experience*/*exploration* values in high *attention*, whereas in low *attention* they are compared with each other.

**Fig. 8:**
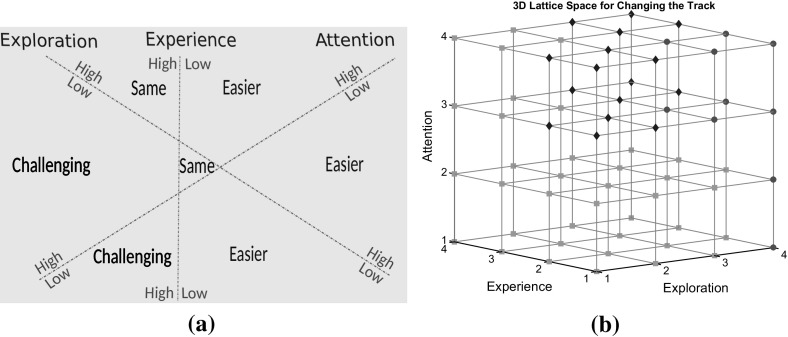
**a** The outcome of the *Segment Altering Decision* algorithm depends on the values of *Exploration*, *Experience* and *Attention* and their assigned thresholds. **b** 3D Lattice of User Model showing the implicit space of the high level variables instructing the algorithm to change a particular segment. *Colour coding* of the decisions is respected between the two figures. (Color figure online)

Giving instructions to change the track at this point is straightforward and is shown in Fig. 8a. In more detail, when there is a high *Attention*, defined through a threshold, and:

1. Both *Experience* and *Exploration* are High: The user is in the optimal region so the segment should be kept the same.
2. Both *Experience* and *Exploration* are Low: The user is engaged since *Attention* is high, therefore we should allow more time for the user to adapt and build up skill so the segment should still stay the same.
3. High *Experience*, Low *Exploration*: A more challenging track should be provided (e.g. one with more or sharper curves) since the user’s skills are high and challenges drop below the threshold.
4. Low *Experience*, High *Exploration*: An easier segment should be created (e.g. one that imitates a previous user path, or with less curvature) since the user’s skills cannot cope with the current challenge of the path.

When the algorithm detects low *Attention*, *Experience* and *Exploration* values are compared to themselves and not to a threshold, therefore:

5. *Experience* larger than *Exploration*: Skills are greater than the challenges so the path should become more challenging.
6. *Experience* lower than *Exploration*: Skills are lower than the challenges of that path so an easier path should be provided.
7. *Experience* same as *Exploration* (both low): Both values are low and *Attention* of the user is also low therefore an easier segment should be provided.

Using the above information and Fig. 8a we constructed a 3D lattice, shown in Fig. 8b, that covers the implicit space of those three variables. Each region in the lattice (indicated by the different colours/shapes) instructs the algorithm to either leave the segment untouched (blue—diamond) or change the segment to either an easier (green—square) or harder (red—circle) path according to the user model.

In order to plot the lattice, we used a 50% threshold for each class in order to define high and low values. Our user model consists of various parameters and thresholds (e.g. proportion *jump*—*J*) that can be altered as user spends more time with the game. In this article we are not focusing on how these parameters are changing the outcome of the algorithm, however, the reported values (see Table 10) are the ones used to derive the results of our experiment. Their values were chosen to mid-percentage or common values regarding the variability of the subjects collected, although there might be more optimal ones.

Specifically, the two variables representing the “jump” ($J^s_k$, $^{p}J^s_k$)—amount of improvement of the user in respect to their average experience—were set at 20% as this was found empirically to be a good proportion of improvement. The thresholds relating to the experience ($^{np}T$, $Experience_T$), were set at the median of the reported experience of our participants, so that the algorithm would create a good distribution of all the outcomes. The thresholds deciding for high/low exploration ($Exploration_T$) and attention ($Attention_T$) were set at 50% (middle range) as both of their definitions (*Transformation Rules*) were designed to give values equally in the negative and positive region.

**Table 10:** Values of the various *thresholds* and *parameters* of the model

Variable	Percentage
1. $J^s_k$	20
2. $^{p}J^s_k$	20
3. $^{np}T$	40
4. $$ Experience _T$$	40
5. $$ Exploration _T$$	50
6. $$ Attention _T$$	50

For each segment *s* and each metric *k* is possible to use a different value, however in order to retrieve our results we used the same for all segments and metrics

## Results

The simulator has been driven by 52 users and all of the data were recorded and analysed offline. In the following section we conducted a user profiling analysis in order to verify and find the patterns emerging from our user responses to determine our user types. We also validated our *Framework*’s outcomes using the data collected from the users through their responses.

### User profiling

An outstanding user model needs to be compatible with the user’s game play experience. The latter depends on the type of the player as well. Type can be subcategorised into multiple groups, like regular gamers versus non-gamers or/and racing enthusiasts or not, etc. As we noticed from our experiments, the users who were involved in racing communities were more willing to do the experiment, they were better engaged and they have more constructive feedback than novice. A successful user model is to detect these kinds of patterns and act according to the individual’s conceptions.

For better understanding of the different categories of our subjects, we performed user profiling on the questionnaire of our experiment. As shown in Table 11, we collected data from subjects of various groups (e.g. age, gender, racing gamers and real driving experience) so that we can implement a user model that can adapt to diverse player types.

**Table 11:** User demographic profiling: static (Nos. 1–5) and game specific (Nos. 6–10) information analytics regarding the users in our experiment

Analytics from 52 subjects	Results
1. Age	19–35 ($$M = 25$$, $$ SD = 4$$)
2. Driving years	0–18 ($$M = 5$$, $$ SD = 5$$)
3. Gender	87% men, 13% women
4. Driving license	83% yes, 17% no
5. Game play frequency	31% never, 52% occasionally, 15% frequently, 2% every day
6. Track difficulty	4% easy, 54% medium, 38% hard, 4% very hard
7. Improvement	1 nothing, 27 little, 14 a lot, 10 very much
8. Fatigue	24 nothing, 21 little, 6 a lot, 1 very much
9. Pre self-rating	21 beginners, 18 intermediates, 12 advanced, 1 expert
10. Post self-rating	25 beginners, 18 intermediates, 9 advanced

We collected data from 52 subjects of broad range of ages, both genders and various skills in real driving and car game racing

From analytics Nos. 9 and 10 of Table 11, which reports the score that the users gave to themselves before and after the experiment, we noticed (as expected) a higher population in the lower scores since most of our subjects were not frequent racing game players (No. 5). From data obtained from analytic No. 8, we can also infer that most of the users enjoyed the game and didn’t feel tired through the process. We acknowledged from the user’s feedback that the low score on tiredness was either because 20 laps were not enough for them to become fatigued or the subjects were very excited to try a racing simulator. Likewise, the improvement (No. 7), was mostly minor since large sample of the users were either beginners or intermediates, therefore they couldn’t improve much in such a short time, considering that any pre-training was not provided.

Analytic No. 6 is the score of the track on the factor of difficulty. Our aim was to provide a starting track that was in general not very challenging but also not particularly easy. Most of the scores lie in the middle range therefore, our objective was accomplished. However, user feedback disclosed that the particular question might not have been answered as initially envisaged. Subjects were supposed to respond with a level of difficulty regarding their performance. Instead, some responded by taking into account only the appearance of the track’s path and length.

**Fig. 9:**
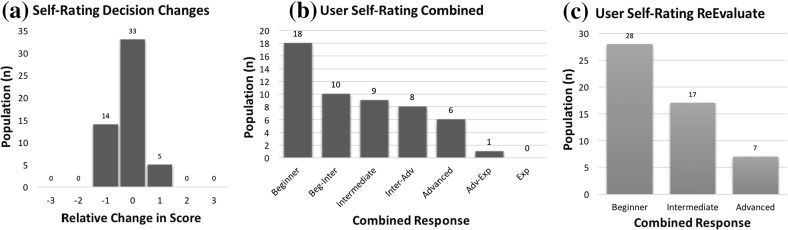
37% of the users changed their decision by only one level during self-rate before and after the experiment. Therefore, to be able to retrieve combined results from both of their responses, we re-evaluate each user into three groups

Some users commented on the fact that there should have been more categories to rate themselves, especially towards the higher range. Also, we notice that only 37% of the users changed their skills rating by a level, as shown by Fig. 9a. Specifically, subjects that lowered their scores commented that they didn’t perform as well as they expected. Since the change was only by a level and most of the users haven’t altered their score, we decided to combine the two (pre and post) responses. By adding the ordinal values of the two *Self-Rate* (*Pre* and *Post*) responses we created a much elaborated description of our users (see Fig. 9b). However, since eight groups are far too many for the number of our subjects we decided to re-evaluate them into three (see Fig. 9c). People who changed their mind were re-evaluated to their lower choice (*self-assigned skill*).

Kruskal–Wallis test between the two *Self-Rate* and *Re-evaluate* skill groups (*Pre* versus *Re-evaluate*, *Post* versus *Re-evaluate*) gave very low *p* values ($p < 0.01$). Therefore we can state that the assigned groups are still dependent to the users’ self-reported skill. For the rest of our results section we are using the *Re-evaluate* skill groups (see Fig. 9c) to correlate the characteristics of the *Framework* and user responses.

The next step is to find particular patterns in the users’ responses and further understand their profile type. The game specific questions labelled as: *Frequency, Fatigue, Difficulty and User Self-Rate (Re-evaluate)* were tested with each other using *Kruskal–Wallis* test.

**Fig. 10:**
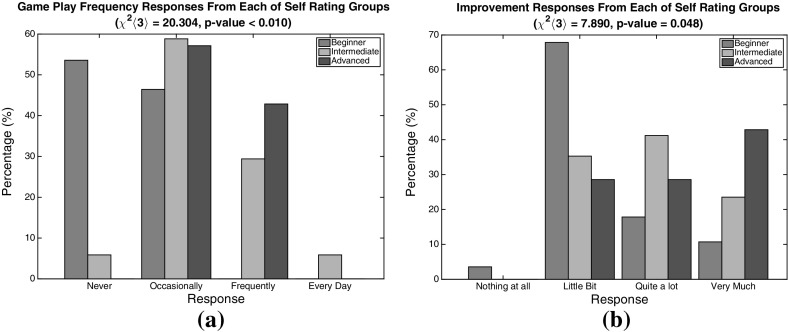
**a** Game play Frequency responses versus *Self-Rate* groups give statistically significant results using Kruskal–Wallis test (*p* value ${<}0.01$). **b** Improved responses versus *Self-Rate* groups give statistically significant results using Kruskal–Wallis test (*p* value ${<}0.05$)

Low *p* value ($p < 0.01$) was observed between the *Self-Rate* response with game play frequency response. As anticipated, people who tend to play racing games frequently are expected to be Intermediate or Advanced whereas those that don’t, will rate themselves as Beginners. Under a pairwise test, there is no distinct separation in the middle-upper classes (e.g. *Frequently* versus *Occasionally*, *Intermediate* versus *Advanced*). Responses from each self-rate group towards game play frequency response are shown in Fig. 10a.

An interesting correlation was also detected between Self-Rate and Improved responses with $p < 0.05$. Beginners tend to respond that their improvement was little to nothing. Whereas most of the Intermediate and Advanced users responded with high improvement as show in Fig. 10b. This can be explained using the ZPD. Players with the required skills and experience could learn on every lap repetition and improve themselves up to their ADL, which is in turn much higher than the player’s marked as *Beginners*. Therefore, assuming all users start at scoring their improvement from the same point then more experienced users will improve more than the rest.

Apart from those mentioned, there were not any other statistically significant results between the responses. Therefore, we can assume from the responses that are not mentioned [*track difficulty* ($\chi ^2(3) = 2.565$, $p = 0.46$) and *fatigue* ($\chi ^2(3) = 6.772$, $p = 0.0795$)] that they cannot be directly associated with the user’s *Self-Rate* response and the user profile in general, however, they can be retrieved during game play. In fact as we will show later, responses concerning *fatigue* are correlated to the user *attention* calculated by our *Framework*.

### Algorithm’s validation

It is important to mention that the algorithm for finding the metrics’ significance (weights) for the *expert*’s model used data from only after the fifth lap, since feedback from the users suggested that familiarity of the track and simulator in general happened after 5–8 laps.

What follows in the rest of this section is the validation of the algorithm’s high level variables and outcomes through the user responses:

*Algorithm’s outcome variability and user’s self-rate response* Chi-squared test was applied to assess the goodness of fit for the variability of the algorithm’s output on the users’ level of expertise. Users were split into three groups according to their combined self-rating response mentioned in Sect. 4.1. For each segment of the track the algorithm evaluated the user through the model, and gave an output for the alteration that has to be made on that segment; this can be either *easier*, *same* or *challenging*. These were set as the category names of the Chi-square table.

By counting the number of category outputs generated from all segments for each of the three groups, we create the Chi-square matrix shown in Table 12. The *null hypothesis* states that the decisions for each group are being drawn from the same distribution and that categories are independent from the groups. The test rejects the null hypothesis with a Chi-square statistic at ${\chi }^2 = 72.19$ and $p < 0.01$. This shows that the algorithm provides different proportions of segment alterations to particular groups.

**Table 12:** Chi-square table of algorithm variability (*expert* model) versus users reported skill shows statistically significant results (${\chi }^2 = 72.19, p < 0.01$)

Group: categories	Easy	Same	Challenging	Total
Beginner	67 [42.54]	68 [77.54]	5 [19.92]	140
Intermediate	12 [25.83]	57 [47.08]	16 [12.10]	85
Advanced	0 [10.63]	19 [19.38]	16 [4.98]	35
Total	79	144	37	260

Number in the squared parentheses indicate the expected value of each group in the particular category

**Fig. 11:**
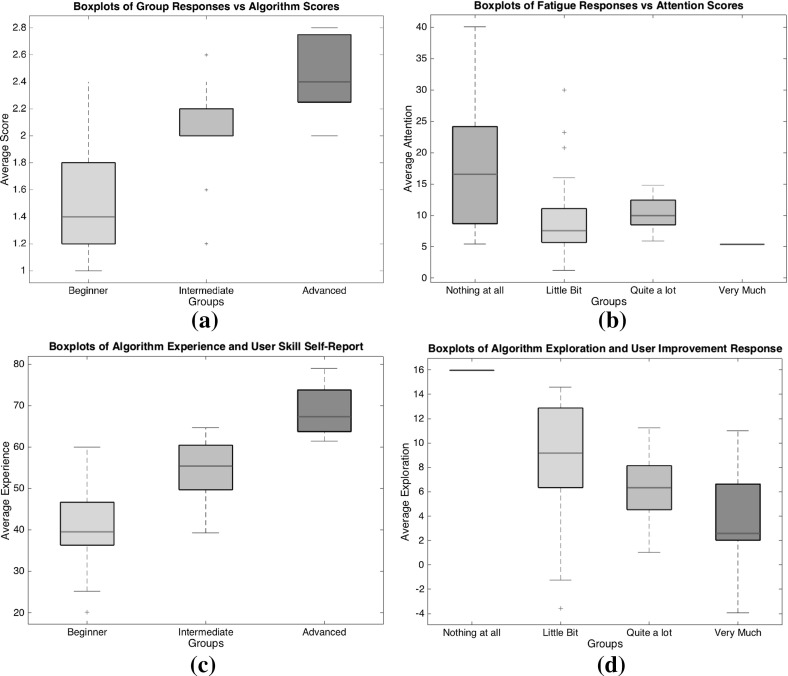
**a** *Boxplots* of the average score of *track altering decision* of each user against their *skill* response. Kruskal–Wallis test shows statistically significant results: ${\chi }^2(2) = 22.64$, $p < 0.01$. Whereas Spearman Correlation: $\rho = 0.66$, *p* value ${<}0.01$ shows a strong positive correlation. **b** *Boxplots* of the average score *Attention* of each user against their response on *fatigue*. Kruskal–Wallis test shows statistically significant results: ${\chi }^2(3) = 11.35$, $p = 0.01$. Spearman Correlation detects a negative correlation: $\rho = -0.41$, $p < 0.01$. **c** *Boxplots* of the average score on *Experience* of each user against their response on self-reported *skill*. Kruskal–Wallis test shows statistically significant results: ${\chi }^2(2) = 28.81$, $p < 0.01$. Spearman Correlation gives a strong positive correlation: $\rho = 0.75$, $p < 0.01$. **d** *Boxplots* of the average score on *Exploration* of each user against their response on *improvement*. Kruskal–Wallis test shows statistically significant results: ${\chi }^2(3) = 12.54$, $p < 0.01$. Whereas Spearman correlation shows a negative correlation: $\rho = -0.48$, $p < 0.01$

Furthermore, by assigning a score value for each category (*easy*: 1, *same*: 2, *challenging*: 3) we calculated the average score for each user from the generated outputs. Utilising Kruskal–Wallis test with the *null hypothesis* that the scores of each group arise from the same distribution gave ${\chi }^2(2) = 22.64$ and *p* value ${<}0.01$. Therefore, there is enough evidence to reject the *null hypothesis*. Box plots of the average score against the groups are shown in Fig. 11a. In addition, as expected, the Spearman correlation between the average score and the user’s skill report is positively correlated ($\rho = 0.66$, $p < 0.01$).

As a result, the tests support the fact that the algorithm generates outputs according to the different levels of expertise of each user. Over the subsequent paragraphs, we will present in more depth the correlation of the users’ responses to the specific metrics of our framework:

*User fatigue and attention metric* The users were asked if they *“felt tired during the experiment because of the lap repetition?” (Question: Fatigue)*. These responses were paired with the algorithm’s average Attention value of each user. Using Kruskal–Wallis test (${\chi }^2(3) = 10.75$, $p = 0.01$) we found that the Attention value is significantly different for different levels of self-reported fatigue (see Fig. 11b). The Spearman correlation test ($\rho = -0.39$, $p < 0.01$) showed that there is a significant negative correlation between Attention value and user’s fatigue response (lowest rank is set to “Nothing at all” whereas “Very Much” is the highest). From this we can infer that the algorithm’s Attention output is also statistically correlated with the self-reported fatigue.

*User experience report and experience metric* Similar to the previous test, the average value of the model’s experience variable of each user was paired to the user’s combined self-rate response. The Kruskal–Wallis test (${\chi }^2(2) = 28.81$, $p < 0.01$) indicates that the average experience score is significantly different for each distinct self-rate skill response (see Fig. 11c). The Spearman correlation test showed a strong positive correlation between experience value and user reported skill ($\rho = 0.75$, $p < 0.01$).

*User improvement report and exploration metric* The average value of the model’s exploration variable of each user was paired with the user’s self-rated improvement. The Kruskal–Wallis test (${\chi }^2(3) = 12.54$, $p < 0.01$) indicates that exploration score is statistically different for the various levels of self-rate improvement (see Fig. 11b). In addition, the Spearman correlation test found a negative correlation between exploration value and self-rated improvement ($\rho = -0.48$, $p < 0.01$).

Furthermore, in Sect. 4.1 we noticed that users with higher skill tend to respond on the improvement question positively. However, results from this test show that subjects with higher improvement response have a lower exploration (challenge) value. This fact reinforces the method of evaluating the user exploration in the algorithm since users with higher skill have less challenges, as the game environment stays constant, and therefore generate lower exploration values.

### Expert–user best models comparison

The results of the previous Sect. 4.2 and the implementation of the user model in Sect. 3.4 mainly focus on comparison of user data with the *expert*’s. However, having expert data for a particular track contradicts the concept of creating new tracks that are tailored to the user. The *Framework* will not be able to model the user when moving from a newly generated segment path to a subsequent segment since expert data will not be available to create the metrics.

In order to confront this issue, on the second level (*Performance Metrics*) of our user model (see Fig. 1), we allow the comparison of the data either with an *expert* or *user’s* “*best*” data. Both comparisons should generate similar results to the higher level of the model. In this section we test this hypothesis by using the model through *user’s* “*best*” data to create *segment decision changes* as in Sect. 4.2 and compare against them.

Therefore, the algorithm was set to provide segment outputs after the completion of 20 laps from each user and the performance metrics were created using comparisons from the *user’s* “*best*” data. The model’s linear weights are trained only on the user’s available data. It is expected that the model will refine itself to the particular user after a number of laps.

**Fig. 12:**
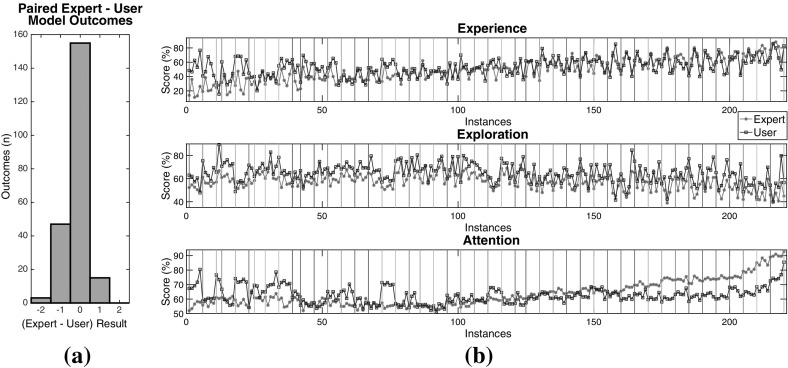
**a** Comparison of the ordinal outputs between models using expert and user data comparisons. Wilcoxon signed rank (two-tailed) test gives $$ Z = -4.417$$ and *p* value $${<}0.01$$, as some of the outcomes shifted towards higher levels. **b** Outputs of high level variables of the two models using *expert* and *user’s* “*best*” data comparisons. The boundaries between the vertical lines indicate the results from different users. For all the variables and for each user, the trends are similar (some differ with a fixed bias per user). Therefore, the difference in results is because the model is overconfident for the abilities of each user and the particular thresholds chosen

The segment decisions of the algorithm were compared with the ones obtained from the *expert* model. Results show that most of the decisions were the same (70.5%) and 29.5% differ mostly towards higher levels: *same* and *challenging* (see Fig. 12a). Using the *Wilcoxon signed rank (two-tailed)* test between paired decisions of the two models, showed that there was a statistically significant change of outcomes between *expert* and *user’s* “*best*” models ($$ Z = -4.417$$, $$p < 0.01$$). The result gives enough evidence to assume that there was a shift towards higher outcomes.

This increment is expected mainly for two reasons. Individual user models might:

1. Not have enough and valid data to create a good model for the user.
2. Be overconfident since the user might perform well when compared to the *user’s best* performance but badly when compared to an *expert*.

Figure 12b shows the values of the three abstract variables, of the *Theoretical Framework* level, that are used to generate the output decisions via thresholds. The vertical bounds indicate results from different users. In general, the sequences between *expert* and *user’s best* are similar but we can identify particular users for whom the two models do not agree (this is mainly because of the second reason mentioned). In future work we will tackle this issue by personalising the thresholds (see Table 10) for each individual user (in the *user’s best* model case) so that the two models are coherent.

As executed in Sect. 4.2, we also performed a Chi-squared test to assess the goodness of fit, for the variability of the algorithm’s output on the user’s combined self-reported skill. By counting the number of category outputs generated from all segments for each of the three groups, we created the Chi-square matrix shown in Table 13. The Chi-square test indicated that depending on the user’s skill group, the model outputs different proportion of a particular segment decision, with statistically significant results (${\chi }^2 = 37.34$, $p < 0.01$).

**Table 13:** Chi-square table of algorithm variability (*User* “*best*” model) versus users reported skill give statistically significant results (${\chi }^2 = 37.34$, $p < 0.01$)

Group: categories	Easy	Same	Challenging	Total
Beginner	30 [18.84]	71 [71.27]	11 [21.89]	112
Intermediate	7 [13.79]	57 [52.18]	18 [16.03]	82
Advanced	0 [4.37]	12 [16.55]	14 [5.08]	26
Total	37	140	43	220

Number in the squared parentheses indicate the expected value of each group in the particular category

**Fig. 13:**
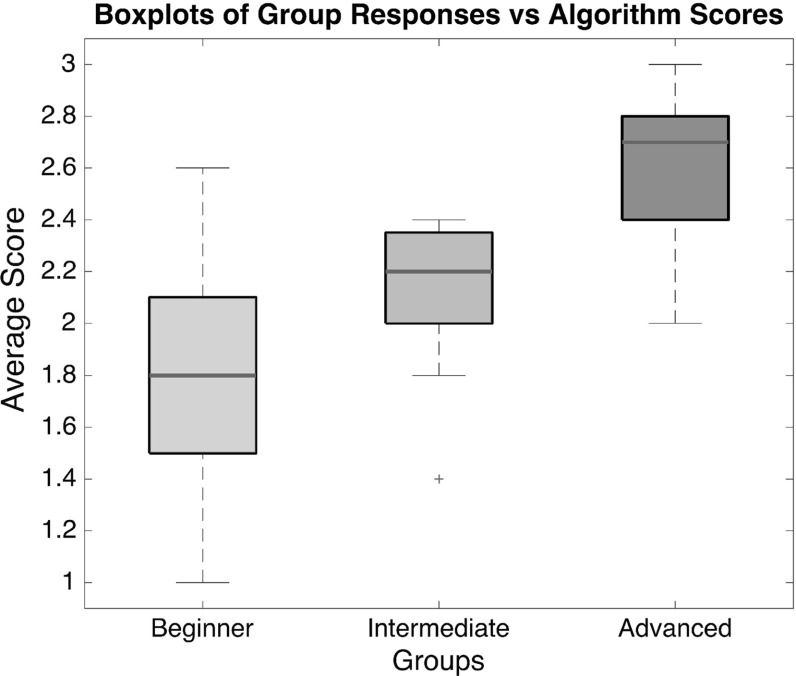
*Boxplots* of the average score of track altering of each user against their skill response. Kruskal–Wallis test shows statistically significant results: ${\chi (2)}^2 = 17.67$, $p < 0.01$. Spearman correlation is strongly positive: $\rho = 0.61$, $p < 0.01$

In addition, the *Kruskal–Wallis* test between the average score obtained for each user from the generated ordinal output decisions and the user’s combined self-reported skill gave ${\chi (2)}^2 = 17.67$ and $p < 0.01$. This shows that the generated scores are a statistically different for different levels of self-reported skill. This can be inferred from Fig. 13 that *advanced* users are well separated from *intermediates* and *beginners*. However, *intermediates* and *beginners* share some overlap. The Spearman’s correlation supports a positive correlation of $\rho = 0.61$ and $p < 0.01$. This correlation is similar to the *expert’s* model segment decision score versus skill comparison ($\rho = 0.66$) result, despite the fact that there is a shift towards higher outcomes for some of the users.

## Discussion

Our objective is to enhance the gaming experience by modelling the user using behavioural and unobtrusive physiological data, and customising the designed tracks in a racing game. By utilising theoretical frameworks on learning and development such as the concepts of *flow* and Zone of Proximal Development, we aim to “keep the player satisfaction at a high level” (Tognetti et al. 2010b). Our user model consists of six processing levels where sequential data from various sensors, user inputs and game outputs are being transformed into theoretical concepts utilised to explain the development of human skills, challenges and attention in that particular task (car racing). *Low level primitives* were designed in a way so as to constitute specific as well as general user characteristics which are then compared to an *expert* value or *user’s best* performance. Each metric is validated for significance using correlation tests whereas the output of the *Framework*’s performance is confirmed using two different techniques: (1) User satisfaction and feedback through questionnaire; (2) by generating a user specific outcome associated with user responses. By applying several statistical tools, we associate the user responses to specific algorithm variables and outcomes to confirm that the algorithm is both in-line with users perspective and with the assumptions we made to design the model. By rejecting the *null hypothesis* in each of our tests in Sect. 4.2 we showed that the model is able to:

- distinguish between the skill level of each user by evaluating the *Experience*
- keep track of the improvement of the user through *Exploration*
- specify the user’s *Attention* through the data
- create personalised outcomes for each particular user which are still conform to their skill group
- function and elicit similar results without using *expert* data
- be further configured through model’s parameters to fit user progress

Compared with similar model approaches specified in Sect. 2 our methodology was not to define the particular features that are correlated to specific user responses and actions in the game, despite the fact that the model implicitly defines feature significance through their weights. Instead, we incorporated all of our available sensory and game data, obtained from each user, into our model and let the algorithm specify their applicability in every situation (i.e. segments). Our hypotheses of different feature significance according to various situations and even between different users are confirmed. The former is specified through the different weights in Tables 7, 8, and 9 along each particular segment.

Backlund et al. (2006) found positive correlations between skill and improvement when students were rated by their instructors in a real driving experiment. In our results we show that we are in agreement with their statement; users that rated themselves and were also defined by the algorithm as advanced drivers seem to be able to improve faster and more than users in the beginner group.

In addition, Togelius et al. (2007) created new tracks that fit the driving style of an agent that was trained on a particular user. They stated that depending on the racing skills of each user (*beginner* vs *advanced*) their generated tracks developed either easier paths (more straight lines, less sharp turns) or harder ones. We showed that the sum of the generation of our ordinal output instructions is correlated to the ordinal level of skills of the user. Therefore, our adaptive user model framework behaves in a similar manner to their findings. Moreover, our model also integrates *attention* and *exploration* concepts in order to describe the current state of the user and adapt better to the individual.

Our future direction is to further validate our hypotheses and the specific variables of the *Framework*. This can be achieved by modelling the users through a multiple track scenario consisting of personalised (*experiment*) and fixed (*control*) transitions of tracks that would allow us to observe the engagement and training through the groups of users.

### Modification and transferability

The proposed method of the adaptive user model is based on having independent hierarchical levels. Thus each level can be modified to fit the requirements of the user activity that needs to be modelled:

- The *non-physiological* data of the *Low Level Primitives* level, such as the game outputs and the user inputs are specific to the task. These can be assigned as anything from mouse clicks to explicit complex actions that are significant to the progress of the competing task.
- For the *Weighting Model* level, the designer should specify the target or a combination of them that provide a measure of global performance to the task (i.e. total score, completion time, number of wins/kills). Moreover, the regression model can be switched with any other similar machine learning technique that provides weights for every input towards a specific output.
- The last three higher levels are interrelated to the *Theoretical Framework* chosen for the task. In our case we are interested in the development of the user’s driving skills and the engagement of the user to the game. From our chosen psychological frameworks, these are evaluated through *Experience*, *Exploration* and *Attention*. Thus there is a need for three transformation rules in the *Trace Theory* level, which are specified by the task designer/expert.
- The *Goal* level utilises the thresholds set on the evaluated high level variables and the outcome of the model depends on the task (i.e. personalisation of the track’s paths).

*Example: Training children with cognitive difficulties to use a wheelchair* Our model could be used to identify particular weaknesses a child possesses when handling a wheelchair—via the model’s weighted metrics—when the *Task* is to drive through a narrow door or performing a turn in a corridor. The *Goal* could be to either alter the scenario to fit the particular user (i.e. increase the door’s opening, make the turn less sharp) or personalise the assistance that gives to the user.

The *Low Level Primitives* as in our case, could consist of user’s driving inputs, eye gaze/fixations, blinks, head pose, wheelchair’s path, velocities and angle, task completion time, collisions etc. Whereas the *Weighting Model* target could be a combination of the path-angle metric, since this is the most important aspect in this task. As this task also involves learning, development as well as engagement of the user and the task involves driving, the *Theoretical Framework* and transformation rules (*Trace Theory* level) could be the ones we already proposed.

### Limitations

The main limitation of the studies we reported in this paper is that the evaluations are based on self-reported user data using Likert-scale self-reports. It is difficult to assess whether responses are reliable or if they are affected by other factors that haven’t been considered. An argument emerges that it is not sufficient to assume that users are capable of understanding their own performance and potential. Therefore, responses tend to be subjective to the particular person and the situation s(he) provides them, and thus can be biased.

However, we addressed this issue by (a) collecting data from many users (52) so that the results are statistically significant and (b) carrying out user profiling tests to first check that our user models are varied and to identify any patterns or irregularities between their responses (Fan et al. 2006). In future work we also plan to add user monitoring when driving new personalised tracks and attempt to validate the implied characteristics of our *Framework* through the direct comparison between the user’s performance and engagement in different trials.

On a more technical detail, we employed linear based models to calculate the weights used to evaluate the high level variables in the *Theoretical Frameworks* level. These weights may not provide optimal values since the metrics might not necessarily be linear to the *Goal* (Time metric) we determined, which will be addressed in future work. Furthermore, the evaluation of the *Attention* variable is based exclusively on a game-determined approach and does not take into account any latent emotional states of the user. In addition, the selection of our sensors influence our model’s operation; we chose sensors that are unobtrusive to the user and the task. However, this can sometimes cause data loss or inaccuracy since they have a finite area of tracking. The user model can be designed to adapt to these problems by continuously re-calculating the weights, although an extended loss or stream of erroneous data can lead to an inadequate user model. In our future work we are planning to employ additional physiological sensors (i.e. heart rate, skin conductance) that will enhance the evaluation of user’s immersion to the task.

## Conclusion

In this article we propose an adaptive user modelling framework for transforming user’s low level primitives composed of game-related user actions, game outputs and unobtrusive physiological data such as head pose and eye tracking into theoretical frameworks of learning and development (e.g. concept of *flow* and Zone of Proximal Development). Our intention is to provide a flexible algorithm that identifies the on-line weaknesses and performance of a race driver so that we can alter the track’s path to fit the individuals level of skill. The functionality of the framework so far is to provide segment alteration instructions (i.e. *same*, *easier*, *challenging*) and also point out the human factors, that lead to the particular decisions. The design and implementation of a new path as well as the experiments regarding that, are outside the scope of this paper.

Real user experiments using a simulator setup allowed us to calculate the correlations of our extracted features (*Low Level Primitives*) and estimate the engagement of the users to the task, through their comparisons to an expert—a trained and self-engaged—user. In addition, simple and logical rules regarding the racing task were embraced in order to perform the Trace Theory’s transformation process of low level traces to the theoretical frameworks. The fitness of the rules and in general the modelling process was verified by associating user responses to the experiment with the offline instructions generated. User profiling helped us understand the variation in the user types, whereas feedback helped us to improve our future experiments. An interesting result is that our user responses were not found to be statistically different between genders as indicated in Backlund et al. (2006).

The future development of the user model is to verify the results through experiments where the track is being altered through the decisions of the model in real time, which will allow the user to drive the new track straight away. User attention, skill and challenges will be monitored with the aim of keeping above a threshold through the whole process. This approach will also validate the *Framework* without the need of user responses. As far as we are aware, this kind of experiment hasn’t been conducted before. However, the forthcoming main challenge is to elicit a new path that fits the user model. The path construction algorithm has to include both the instruction generated and the specific human factors that lead to the decision in order to create new segments that will both improve the *engagement* and assist the *training* of the user.

An important future aim will be the adaptation of that model structure to another game or task through minimal changes. This will prove the transferability of the model with as few modifications possible and show its generic capabilities to capture the engagement and enhance user training.

## Footnotes

1 rFactor2 Simulator: http://rfactor.net/web/rf2/.

2 Vision Racer VR3: http://www.visionracer.co.uk.

3 Klotski is a sliding block puzzle where users slide blocks through the confined space in order to move the main block through the exit door.

## References

[CR1] Backlund, P., Engström, H., Johannesson, M.: Computer gaming and driving education. In: Proceedings of the Workshop Pedagogical Design of Educational Games Affiliated to the 14th International Conference on Computers in Education (ICCE), Citeseer, pp. 9–16 (2006)

[CR2] Blanton ML, Westbrook S, Carter G (2005). Using Valsiner’s zone theory to interpret Teaching practices in mathematics and science Classrooms. J. Math. Teacher Educ..

[CR3] Bouvier P, Sehaba K, Lavoué E (2014). A trace-based approach to identifying users engagement and qualifying their engaged-behaviours in interactive systems: application to a social game. User Model. User Adapt. Interact..

[CR4] Breiman L (2001). Random forests. Mach. Learn..

[CR5] Brown, E., Cairns, P.: A grounded investigation of game immersion. In: CHI’04 Extended Abstracts on Human Factors in Computing Systems, pp. 1297–1300. ACM, Vienna (2004)

[CR6] Cardamone, L., Loiacono, D., Lanzi, P.L.: Interactive evolution for the procedural generation of tracks in a high-end racing game. In: Proceedings of the 13th Annual Conference on Genetic and Evolutionary Computation, pp. 395–402. ACM, Dublin (2011)

[CR7] Charles, D., Kerr, A., McNeill, M., McAlister, M., Black, M., Kcklich, J., Moore, A., Stringer, K.: Player-centred game design: player modelling and adaptive digital games. In: Proceedings of the Digital Games Research Conference, Vancouver, BC, Canada, vol. 285, p. 100 (2005)

[CR8] Chen J (2007). Flow in games (and everything else). Commun. ACM.

[CR9] Clauzel D, Sehaba K, Prié Y (2011). Enhancing synchronous collaboration by using interactive visualisation of modelled traces. Simul. Model. Pract. Theory.

[CR10] Costa PT, McCrae RR (1995). Domains and facets: hierarchical personality assessment using the revised neo personality inventory. J. Personal. Assess..

[CR11] Coyne R (2003). Mindless repetition: learning from computer games. Des. Stud..

[CR12] Csikszentmihalyi M (1990). Flow: The Psychology of Optimal Experience.

[CR13] Csikszentmihalyi M (2000). Beyond Boredom and Anxiety.

[CR14] Demiris, Y.: Knowing when to assist: Developmental issues in lifelong assistive robotics. In: Annual International Conference of the IEEE on Engineering in Medicine and Biology Society (EMBC), Minneapolis, US, pp. 3357–3360 (2009) 10.1109/IEMBS.2009.5333182 19964078

[CR15] Doshi A, Trivedi MM (2012). Head and eye gaze dynamics during visual attention shifts in complex environments. J. Vis..

[CR16] Fan X, Miller BC, Park KE, Winward BW, Christensen M, Grotevant HD, Tai RH (2006). An exploratory study about inaccuracy and invalidity in adolescent self-report surveys. Field Methods.

[CR17] Fanelli, G., Gall, J., Van Gool, L.: Real time head pose estimation with random regression forests. In: Computer Vision and Pattern Recognition (CVPR), Colorado Springs, US, pp. 617–624 (2011)

[CR18] Frey BJ, Dueck D (2007). Clustering by passing messages between data points. Science.

[CR19] Galligan, L.: Using Valsiner. In: Proceedings of the 31st Annual Conference of the Mathematics Education Research Group of Australasia (MERGA31): Navigating Currents and Charting Directions, Mathematics Education Research Group of Australasia (MERGA), Brisbane, Australia, pp. 211–218 (2008)

[CR20] Hong JC, Liu MC (2003). A study on thinking strategy between experts and novices of computer games. Comput. Hum. Behav..

[CR21] Hunicke, R., LeBlanc, M., Zubek, R.: Mda: a formal approach to game design and game research. In: Proceedings of the AAAI Workshop on Challenges in Game AI, vol. 4 (2004)

[CR22] IJsselsteijn W, Poels K, De Kort Y (2008). The Game Experience Questionnaire: Development of a Self-Report Measure to Assess Player Experiences of Digital Games.

[CR23] Jennett C, Cox AL, Cairns P, Dhoparee S, Epps A, Tijs T, Walton A (2008). Measuring and defining the experience of immersion in games. Int. J. Hum. Comput. Stud..

[CR24] Kivikangas JM, Chanel G, Cowley B, Ekman I, Salminen M, Järvelä S, Ravaja N (2011). A review of the use of psychophysiological methods in game research. J. Gaming Virtual Worlds.

[CR25] Koster R (2013). Theory of Fun for Game design.

[CR26] Lawson CL, Hanson RJ (1974). Solving Least Squares Problems.

[CR27] Loiacono D, Cardamone L, Lanzi PL (2011). Automatic track generation for high-end racing games using evolutionary computation. IEEE Trans. Comput. Intell. AI Games.

[CR28] MacQueen, J., et al.: Some methods for classification and analysis of multivariate observations. In: Proceedings of the Fifth Berkeley Symposium on Mathematical Statistics and Probability, Oakland, CA, USA, vol. 1, pp. 281–297 (1967)

[CR29] Malone, T.W.: What makes things fun to learn? Heuristics for designing instructional computer games. In: Proceedings of the 3rd ACM SIGSMALL Symposium and the First SIGPC Symposium on Small Systems, pp. 162–169. ACM, London (1980)

[CR30] Malone TW, Lepper MR (1987). Making learning fun: a taxonomy of intrinsic motivations for learning. Aptit. Learn. Instr..

[CR31] Mead JL, Renaut RA (2010). Least squares problems with inequality constraints as quadratic constraints. Linear Algebra Appl..

[CR32] Nakamura J, Csikszentmihalyi M, Snyder CR, Lopez Shane J (2002). The concept of flow. Handbook of Positive Psychology.

[CR33] Settouti, L.S., Prie, Y., Marty, J.C., Mille, A.: A trace-based system for technology-enhanced learning systems personalisation. In: Ninth IEEE International Conference on Advanced Learning Technologies (ICALT), pp. 93–97. IEEE, Riga (2009)

[CR34] Spearman C (1904). The proof and measurement of association between two things. Am. J. Psychol..

[CR35] Steels, L.: The architecture of flow. In: Tokoro, M., Steels, L. (eds.) A Learning Zone of One’s Own, pp. 137–149. IOS press (2004)

[CR36] Togelius, J., Lucas, S.M.: Evolving robust and specialized car racing skills. In: IEEE Congress on Evolutionary Computation (CEC), Vancouver, BC, Canada, pp. 1187–1194 (2006)

[CR37] Togelius, J., De Nardi, R., Lucas, S.M.: Making racing fun through player modeling and track evolution. In: SAB’06 Workshop on Adaptive Approaches for Optimizing Player Satisfaction in Computer and Physical Games, pp. 61–70 (2006)

[CR38] Togelius, J., De Nardi, R., Lucas, S.M.: Towards automatic personalised content creation for racing games. In: IEEE Symposium on Computational Intelligence and Games (CIG), Honolulu, Hawaii, pp. 252–259 (2007)

[CR39] Tognetti, S., Garbarino, M., Bonarini, A., Matteucci, M.: Modeling enjoyment preference from physiological responses in a car racing game. In: IEEE Symposium on Computational Intelligence and Games (CIG), Copenhagen, Denmark, pp. 321–328 (2010a)

[CR40] Tognetti, S., Garbarino, M., Bonarini, A., Matteucci, M.: Modeling player enjoyment from physiological responses in a car racing game. In: Proceedings IEEE International Conference on Computational Intelligence and Games, pp. 321–328 (2010b)

[CR41] Valsiner J (1997). Culture and the Development of Children’s Action: A Theory of Human Development.

[CR42] Valsiner J (2005). Scaffolding within the structure of Dialogical Self: hierarchical dynamics of semiotic mediation. New Ideas Psychol..

[CR43] Vygotsky L (1978). Interaction between learning and development. Read. Dev. Children.

[CR44] Whitton N (2011). Game engagement theory and adult learning. Simul. Gaming.

[CR45] Wolfe J (2008). Annotations and the collaborative digital library: effects of an aligned annotation interface on student argumentation and reading strategies. Int. J. Comput. Support. Collab. Learn..

[CR46] Yannakakis, G.N.: Learning from preferences and selected multimodal features of players. In: Proceedings of the 2009 International Conference on Multimodal Interfaces. ICMI-MLMI ’09, pp. 115–118. ACM, New York (2009)

[CR47] Yannakakis GN, Hallam J (2008). Entertainment modeling through physiology in physical play. Int. J. Hum. Comput. Stud..

[CR48] Yannakakis, G.N., Hallam, J.: Ranking vs. preference: a comparative study of self-reporting. In: D’Mello, S., Graesser, A., Schuller, B., Martin, J.C. (eds.) Affective Computing and Intelligent Interaction (ACII). Lecture Notes in Computer Science, vol. 6974, pp. 437–446. Springer, Berlin (2011)

[CR49] Yannakakis GN, Martínez HP, Jhala A (2010). Towards affective camera control in games. User Model. User Adapt. Interact..

[CR50] Yannakakis GN, Spronck P, Loiacono D, André E (2013). Player modeling. Dagstuhl Follow-Ups.

[CR51] Zyda M (2005). From visual simulation to virtual reality to games. Computer.

